# Hierarchical Neural Circuit Theory of Normalization and Inter-areal Communication

**DOI:** 10.1101/2025.07.15.664935

**Published:** 2025-07-19

**Authors:** Asit Pal, Shivang Rawat, David J. Heeger, Stefano Martiniani

**Affiliations:** 1 Simons Center for Computational Physical Chemistry, Dept. of Chemistry, New York University, NY, USA; 2 Center for Soft Matter Research, Dept. of Physics, New York University, NY, USA; 3 Courant Institute of Mathematical Sciences, New York University, NY, USA; 4 Dept. Of Psychology, New York University, NY, USA; 5 Center for Neural Science, New York University, NY, USA

## Abstract

*The primate brain exhibits a hierarchical, modular architecture with conserved microcircuits executing canonical computations across reciprocally connected cortical areas. Though feedback connections are ubiquitous, their functions remain largely unknown. To investigate the role of feedback, we present a hierarchical neural circuit theory with feedback connections that dynamically implements divisive normalization across its hierarchy. In a two-stage instantiation (V1* ↔ *V2), increasing feedback from V2 to V1 amplifies responses in both areas, more so in the higher cortical area, consistent with experiments. We analytically derive power spectra (V1) and coherence spectra (V1-V2), and validate them against experimental observations: peaks in both spectra shift to higher frequencies with increased stimulus contrast, and power decays as 1/f*^4^ *at high frequencies (f). The theory further predicts distinctive spectral signatures of feedback and input gain modulation. Crucially, the theory offers a unified view of inter-areal communication, with emergent features commensurate with empirical observations of both communication subspaces and inter-areal coherence. It admits a low-dimensional communication subspace, where inter-areal communication is lower-dimensional than within-area communication, and frequency bands characterized by high inter-areal coherence. It further predicts that: i) increasing feedback strength enhances inter-areal communication and diminishes within-area communication, without altering the subspace dimensionality; ii) high-coherence frequencies are characterized by stronger communication (ability to estimate neural activity in one brain area from neural activity in another brain area) and reduced subspace dimensionality. Finally, a three-area (V1* ↔ *V4 and V1* ↔ *V5) instantiation of the theory demonstrates that differential feedback from higher to lower cortical areas dictates their dynamic functional connectivity. Altogether, our theory provides a robust and analytically tractable framework for generating experimentally-testable predictions about normalization, inter-areal communication, and functional connectivity.*

## In brief

A hierarchical recurrent neural circuit theory with reciprocal connections, that dynamically implements divisive normalization, predicts key experimental findings. The theory offers a unified view of inter-areal communication in which both communication through subspace and communication through coherence are emergent properties, predicting that high-coherence frequencies exhibit stronger inter-areal communication and reduced subspace dimensionality.

## Introduction

The primate brain is a complex hierarchical network of interconnected cortical areas that perform computations essential for sensorimotor processing and cognitive functions. Among these, certain computations are repeated across cortical areas and are thus said to be “canonical computations”. Divisive normalization [1, 2] is one such fundamental canonical principle.

Feedback connections are ubiquitous in the primate brain [3–5], yet their precise roles in neural computation and communication remain largely unknown [6–8]. They have been hypothesized to serve various functions, including attentional modulation [9–11], inter-areal communication [12], learning [13, 14], and generative modeling [15]. Although divisive normalization has been extensively studied in feedforward recurrent networks [16–19], its implementation and effects in hierarchical networks with feedback connections remain unexplored.

Effective communication between brain areas is essential for complex cognitive tasks, yet the precise mechanisms of communication remain an active area of investigation. One prominent hypothesis, “communication through coherence” (CTC) [20, 21], suggests that synchronized oscillations (coherence) across brain regions during diverse cognitive, perceptual, and behavioral tasks are crucial for effective inter-areal communication [22–26]. However, the causal nature of the relationship remains a key unresolved question. It is currently debated whether coherence is a fundamental mechanism enabling interareal communication or rather an epiphenomenon arising from it [27–31]. Another prominent alternate hypothesis proposes that brain areas communicate through a low-dimensional “communication subspace (CS)” embedded within the high-dimensional neural activity [32–35]. This concept, also identified in artificial neural networks [36], posits that only activity within these shared subspaces between the source and target areas facilitates communication. Currently, a comprehensive theoretical framework capable of simultaneously exploring these different communication mechanisms and explaining their dynamic modulation by factors such as feedback and stimulus contrast is lacking.

**Figure 1:**
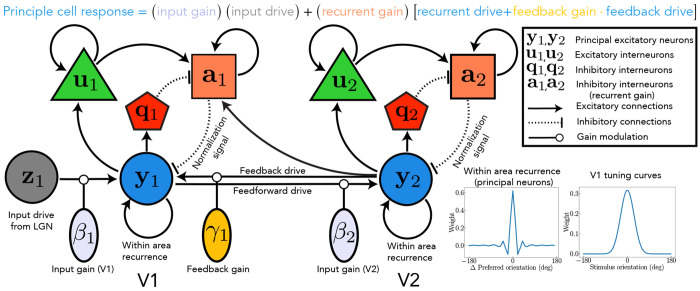
Schematic of the two-stage recurrent circuit model. *Solid and dashed lines represent excitatory and inhibitory connections, respectively. Principal excitatory neurons are denoted by* **y**, *modulatory excitatory neurons by* **u**, *and modulatory inhibitory neurons by* **a** *and* **q**. *Subscripts 1 and 2 designate neurons in visual areas V1 and V2, respectively*. **z**_**1**_ *denotes the input drive from the LGN to V1, a weighted sum of the responses of LGN neurons. Parameters β*_1_ *and γ*_1_ *modulate the input and feedback gain to V1, respectively*.

To address these unresolved questions, we introduce a novel hierarchical recurrent neural circuit theory (Fig. 1) built on two *a priori* constraints: i) a hierarchy of brain areas with recurrent, feedforward, and feedback connections, and ii) recurrently implemented normalization within each cortical area. Based on the theory, we derive analytical expressions to compute inter-areal coherence and communication subspaces, offering experimentally testable predictions about the effects of both input gain and feedback strength on inter-areal communication. As a consequence of these two *a priori* constraints, our theoretical framework gives rise to emergent properties that explain a wide spectrum of neurophysiological phenomena, including modulation of contrast response functions, oscillatory dynamics evident in both power and coherence spectra, communication subspaces, and the dynamic control of functional connectivity. A central contribution of this work is to present a unified view of inter-areal communication. The theory simultaneously predicts empirical observations of both communication subspaces and interareal coherence, and reveals that coherence shapes the communication subspace.

Note that throughout our analysis, we neither adjust model parameters (such as time constants, weight matrices, and noise amplitudes) to reproduce specific experimental trends nor fit data when making direct comparisons. All results presented were obtained using a *single* set of model parameters.

## Results

### Theory

Initially conceived to explain the responses in the primary visual cortex (V1) [1], the normalization model has emerged as a canonical computation across sensory and cognitive domains [2], including higher level vision [37, 38], olfaction [39], audition [40, 41], somatosensation [42], attention [43–55], multisensory integration [56–60], working memory [61–64], and decision-making [65–68]. At its core, the normalization model proposes that the response of an individual neuron is scaled by the collective activity of its neighboring neurons (viz., the normalization pool), analogous to normalizing the length of a vector. Functionally, this computation serves as an automatic gain control mechanism, enabling neurons to operate effectively over a broad dynamic range of inputs [1, 69, 70]. It also simplifies the interpretation of neural signals by downstream brain regions [70–72] and maintains response consistency across different input conditions [1, 39, 69, 70, 73]. In addition, normalization decorrelates population responses to reduce redundancy [39, 74–77], prevents excessive recurrent excitation to maintain network stability, [18, 19], and fosters efficient learning [78, 79].

Normalization is a computational-level description of neural responses that can be implemented using various mechanisms. Experimental evidence suggests that normalization operates via recurrent amplification [80–83], amplifying weak inputs more than strong inputs, consonant with evidence that cortical circuits are dominated by recurrent excitation [84–89]. ORGaNICS is one such theoretical framework that dynamically implements divisive normalization by modulating recurrent amplification [16, 17]. Previous work on normalization models (including previous work on ORGaNICs) has focused on a single neural circuit within a brain region, such as V1, ignoring the abundant top-down feedback connections. Here, we develop a new hierarchical ORGaNICs theory with feedback in addition to feedforward and recurrent connections.

The hierarchical ORGaNICs theory describes neuronal population responses as dynamic processes evolving in a recurrent circuit, modeled by nonlinear differential equations (see Fig. 1 for illustration and Methods for equations). The circuit comprises distinct cell types: principal (excitatory), modulator (excitatory), and modulator (inhibitory) cells (n.b., “modulator ” refers to a multiplicative computation, not necessarily implemented with neuromodulators). Output firing rates of principal cells depend on three terms: 1) feedforward input drive modulated (viz., scaled) by input gain; 2) feedback drive modulated by feedback gain; 3) the sum of recurrent drive and scaled feedback drive, collectively modulated by recurrent gain. Unlike generic E-I models in which all neurons perform the same computation, ORGaNICs assign unique computations to each cell type. Because normalization ensures circuit stability over a broad range of parameters [18, 19], the theory is uniquely well suited to analytical treatment, e.g., to derive closed-form solutions for the power spectral density, coherence, and communication subspaces (see Methods and Appendix).

Each neuron’s input drive is a weighted sum of responses from a population of neurons at lower stages of the hierarchy. For example, LGN responses provide input drive to V1 (**z_1_** in Fig. 1), and V1 responses similarly drive V2 (solid arrow from **y_1_** to **y_2_** in Fig. 1, representing principal neuron responses in each area). This weighting is defined by an embedding weight matrix composed of positive synaptic weights, which specify each neuron’s stimulus preferences, such as orientation selectivity (Fig. 1, V1 tuning curves), analogous to a standard receptive field model. Input gain is determined independently by input modulator cells (*β*_1_ and *β*_2_ in Fig.1).

The feedback drive is a weighted sum of the responses of a population of neurons in one or more brain areas from a higher stage in the hierarchy, e.g., V2 provides feedback to V1 (Fig. 1, solid arrow from **y_2_** to **y_1_**). The feedback drive is specified by a feedback weight matrix. For simplicity, the feedback weights from V2 to V1 were chosen to be the transpose of the feedforward weight matrix from V1 to V2 [15]. For the exact structure of the different matrices, see Fig. 7. The feedback gain from V2 to V1 is denoted by *γ*_1_ in Fig. 1.

**Figure 2:**
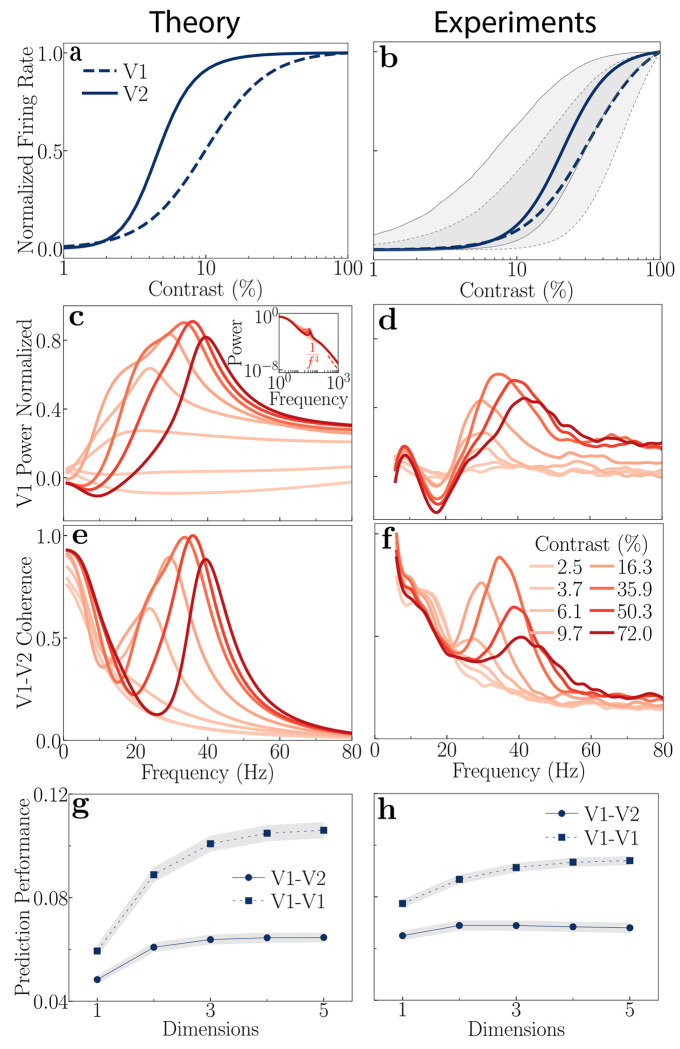
Comparison of theoretical predictions (left column) and experimental observations (right column) in V1 and V2. All theoretical predictions are generated using the same baseline parameters (Table 2). **a,** Theoretical mean firing rates as a function of stimulus contrast (V1: dashed line, V2: solid line). **b,** Experimental mean firing rates of V1 and V2 (data from [90] and [91], replotted for comparison). The shaded area with a solid border indicates the 25th to 75th percentile range for V1, and the one with the dashed border indicates the same for V2. **c,** Theoretical V1 power spectra at various stimulus contrast levels. Power spectra were normalized using the equation $\frac{\left(\text{power}-\text{baseline}\right)}{\left(\text{power}+\text{baseline}\right)}$, where baseline power is taken to be spontaneous activity at 0% contrast. The peak power shifts towards higher frequency with increasing contrast. The inset shows the $\frac{1}{f^{4}}$ power law decay at high frequency. **d,** Experimental power spectra from macaque V1 (data from [22], replotted for comparison). **e,** Theoretical coherence spectra between maximally firing neurons in V1 and V2. The peak coherence shifts towards higher frequency with increasing contrast. **f,** Experimental coherence spectra from macaque V1-V2 (data from [22], replotted for comparison). **g,** Theoretical communication subspaces, prediction performance as a function of dimensionality for inter-area (circles) and within-area (squares) communication. Simulation results indicate that the inter-area communication subspace is lower dimensional than the within-area communication. **h,** Experimental communication subspaces from macaque V1-V2 (data from [32], replotted for comparison). The plotted prediction performance in both panels **g** and **h** is an average across different subsets of the source and target neural populations, and the shaded areas represent the standard error of the mean (SEM).

The recurrent drive is a weighted sum of principal cell responses (Fig. 1, curved arrows from **y_1_** onto itself and from **y_2_** onto itself). For the results reported in this paper, the recurrent weights were selected to have a center-surround architecture in which the closest recurrent connections are excitatory and the more distant ones are inhibitory (Fig. 1, recurrent synaptic weights). The recurrent gain depends on the responses of recurrent modulator cells (**a**_1_ and **a**_2_ in Fig. 1), which in turn depend on: 1) the principal cell responses (**y_1_** and **y_2_** in Fig. 1) via excitatory (**u**_1_ and **u**_2_ in Fig. 1) and 2) inhibitory (**q**_1_ and **q**_2_ in Fig. 1) interneurons; 3) feedback from principal cells at a higher stage (solid arrow from **y_2_** to **a**_1_ in Fig. 1). Normalization is achieved dynamically through the action of the modulator cells, which carry the “normalization signal”.

### Contrast response functions

The two-stage recurrent model with feedback implements divisive normalization exactly, across multiple cortical areas simultaneously (i.e., the model’s steady-state responses align precisely with the analytical steady-state solutions given by the normalization equation Eq. 2 when all the gains are set to *γ* = *β* = 1 and the recurrent weight matrix is identity, see Methods and Appendix). As shown in Fig. 2a, both V1 (dashed) and V2 (solid) exhibit the characteristic saturating, sigmoidal dependence on contrast. The model predicts a lower semisaturation constant (the contrast at which the firing rate reaches half-saturation) and a steeper slope for cortical area V2 compared to V1. This prediction is commensurate with experimental observations in the literature that used optimal stimulus sizes (Fig. 2b) [90, 91]. Similar hierarchical trends, specifically an increase in slope and a decrease in the semisaturation constant, have also been observed across other brain areas like the LGN, V1, and MT with progression up the visual hierarchy [92].

**Figure 3:**
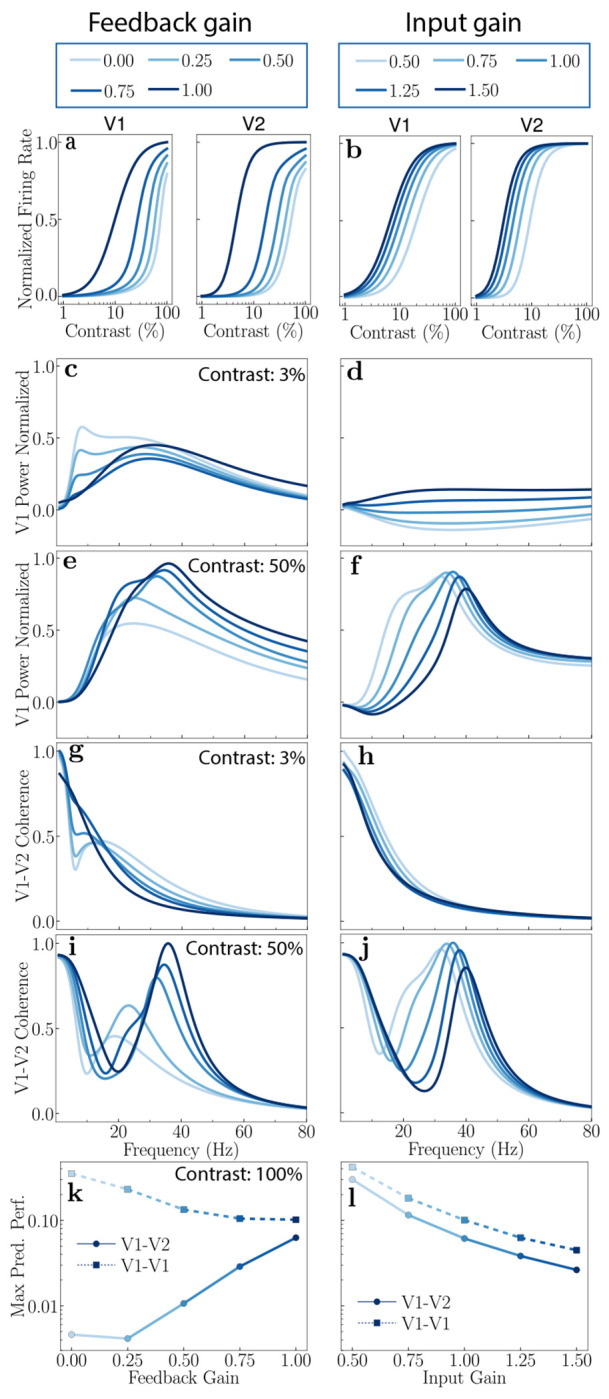
Theoretical predictions: Modulating feedback (left) and input gain (right). **a,b,** Firing rates as a function of contrast. Increasing either feedback gain or input gain enhances neural responses, with feedback gain showing a greater impact in the higher cortical area V2. **c,d,** V1 power spectra: 3% contrast. An alpha peak is observed for low feedback gain, but the peak diminishes with increasing feedback gain. The alpha peak is absent with input gain changes. **e,f,** V1 power spectra: 50% contrast. A consistent gamma peak is observed, which shifts toward higher frequencies with increasing feedback gain (**e**) and input gain (**f**). **g,h,** Coherence spectra: 3% contrast. A broad peak in the beta band is observed at low feedback gain, which vanishes at high feedback gain. No such peak is observed with changes in input gain. **i,j,** Coherence spectra: 50% contrast. A beta peak is observed for low contrasts, which shifts toward higher (gamma) frequencies with increasing feedback gain. No beta peak is observed for changes in input gain, but the gamma peak shifts toward higher frequencies with increasing input gain. **k,l,** Communication subspaces. Increasing feedback gain enhances inter-areal (circles) communication while decreasing within-area (squares) communication. Conversely, increasing input gain decreases both inter- and within-area communication.

Increasing the feedback gain, *γ*_1_, enhances neural responses across cortical areas (Fig. 3a). However, this enhancement is not uniform across cortical areas. The gain change is notably higher for neurons in the higher cortical area (V2). Elevating input gain, *β*_1_, also enhances neural responses across both cortical areas (Fig. 3b). However, unlike the effects observed with feedback gain modulation, the response enhancement from changing input gain is nearly identical in both cortical areas.

### Power Spectra

Power spectra analysis of electroencephalographic (EEG) and local field potential (LFP) signals has been used extensively to identify and quantify oscillatory activity in various frequency bands (e.g., alpha, beta, gamma, theta), hypothesized to be linked to various cognitive functions, behavioral states, and neurological conditions [93–96]. Given that our model has a fixed point solution and is analytically tractable, we can calculate the power and coherence spectra analytically, given a model of the noise in the circuit (see Methods).

We adopted an analytically tractable noise model that adds low-pass filtered synaptic noise to the membrane potential and multiplicative Gaussian white noise to the firing rate dynamics. Furthermore, we explicitly accounted for long-range correlated noise in LFP measurements. This type of noise, propagating through brain tissue, affects LFPs with negligible impact on spiking activity and firing rates [97–99]. Further details are provided in the Appendix (“Intrinsic neural circuit noise” and “Extrinsic noise in the local field potential”).

Our theory successfully replicates key experimental observations of V1 power spectra across varying contrast levels. These theoretical V1 predictions (Fig. 2c) are compared with experimental spectra from macaque V1 (Fig. 2d) under identical contrast conditions [22]. The model accurately captures three critical experimental trends: 1) the power spectrum of the noise is shaped by recurrent normalization to exhibit a resonant peak in the gamma-frequency range (≈ 40-60 Hz); 2) the peak frequency shifts rightward with increasing contrast; and 3) the peak power decreases after reaching a maximum around 40% contrast [100–102]. The oscillatory activity in the gamma-frequency range depends on normalization; removing normalization eliminates the peaks (see Suppl. Fig. 11e). The peak frequencies depend on the values of the intrinsic time constants (see Table 1); larger time constants would shift the peaks to lower frequencies (see Discussion).

The theory also reproduces the steep fall-off in power reported at higher frequencies [103], where power approximately scales as $1/f^{4}$ with increasing frequency ( *f* ) (Fig. 2c, inset). This occurs because the synaptic noise added to the membrane potentials is first low-pass filtered by the synapse’s own time constant, and then by the network model itself that acts as a low-pass filter (each step contributing a factor of $1/f^{2}$).

Modulation of feedback and input gains elicits distinct changes in the power spectra for a constant stimulus contrast. Figures 3c–f illustrate these distinct effects on the normalized power spectra when feedback gain (left panels) or input gain (right panels) is varied. These comparisons are presented for both a low (3%; Figs. 3c,d) and a high (50%; Figs. 3e,f) stimulus contrast. See Supplementary Material for comprehensive results across other contrast levels (Suppl. Fig. 8).

The model predicts oscillatory activity in the alpha-frequency range (≈ 10 Hz) for low stimulus contrasts and small feedback gain values (Fig. 3c). As the feedback gain to the excitatory neurons increases, this alpha peak diminishes and ultimately vanishes at high feedback gain levels, while a broad peak concurrently emerges in the gamma frequency range (Figs. 3c,e). No such oscillatory activity in the alpha-frequency range is observed by manipulating the input gain, for any contrast (Figs. 3d,f).

The power spectra are dominated by oscillatory activity in the gamma-frequency range at high stimulus contrast (Figs. 3e,f). Increasing the feedback gain causes this gamma peak to shift towards higher frequencies. A similar shift of the gamma peak to higher frequencies is observed when the input gain is increased. However, a key distinction arises in their impact on the peak’s bandwidth: the gamma peak bandwidth narrows with increasing input gain but broadens with increasing feedback gain.

### Coherence

Coherence measures the statistical correlation between two signals (e.g., LFPs in two different brain areas) as a function of frequency. Neuronal synchrony is hypothesized to contribute to the dynamic selection and routing of information both within and between brain areas [21, 104, 105].

Our model’s predictions for V1-V2 coherence spectra accurately capture key experimental trends observed across various stimulus contrasts. We analytically determined the coherence between the maximally firing neurons of V1 and V2 (see Methods). Figure 2e displays the theoretically predicted V1-V2 coherence spectra for different contrasts. These model predictions are compared with experimental V1-V2 coherence data from macaque monkeys, obtained under identical contrast conditions (Fig. 2f; [22]). The model accurately captures several crucial experimental observations: 1) high coherence at low frequencies; 2) a shift of the peak coherence toward higher frequencies with increasing contrast; and 3) a decrease in peak coherence after reaching a maximum around 40% contrast.

Feedback and input gain differentially modulate V1-V2 coherence across contrasts (Fig. 3g–j). At low contrast (3%), a coherence peak in the beta band (12-30 Hz) is observed with low feedback gain (Fig. 3g); this peak disappears as feedback gain is increased. Notably, at low feedback gain, a dip in V1-V2 coherence occurs in the alpha band, corresponding to the frequencies where peak power is observed in V1. This suggests that alpha band activity, despite its prominence in V1 power, may not effectively propagate to V2 or participate in inter-areal communication. No such peaks are observed with changes in the input gain for low contrast (Fig. 3h). At high contrast (50%), increasing feedback gain shifts the peak coherence towards higher frequencies and increases both its magnitude and width (Fig. 3i). A similar frequency shift in peak coherence occurs with increasing input gain, but its magnitude and width decrease (Fig. 3j).

### Communication subspaces

Neural communication is hypothesized to be channeled through “communication subspaces (CS)” [32–35]. Although neural activity space is highdimensional, neural communication may not utilize this vast space. CS are lower-dimensional manifolds within the broader neural activity space that preferentially engage in inter-areal communication. In contrast, within-area communication utilizes a distinct, higher-dimensional “private” subspace [32].

Our theory successfully captures key characteristics of the CS (Figs. 2g,h). We derived an analytical model to examine both inter-areal (V1-V2) and within-area (V1-V1) communication (see Methods). We found that the CS is low-dimensional, much lower than the number of neurons (30 neurons for the analysis) in each brain area. In addition, the inter-areal communication (V1-V2) subspace is lowerdimensional than the within-area (V1-V1) communication subspace. Figure 2g illustrates the model prediction performance (i.e., the ability to predict activity fluctuations of a target subpopulation from the activity fluctuations of a source subpopulation using a linear model) as a function of the dimensionality of the CS. These theoretical predictions align well with experimental data (Fig. 2h; [32]).

Feedback and input gain differentially modulate communication subspaces (Fig. 3k,l). Increasing feedback gain from the higher cortical area (V2) to the lower cortical area (V1) enhanced inter-areal communication while diminishing within-area communication (Fig. 3k), without substantially altering the subspace dimensionality (see Supplementary Material, Fig. 10a,b). Conversely, increasing input gain reduced both the inter-area and within-area communication (Fig. 3l). These results are for 100% contrast; see Supplementary Material (Fig. 10c) for other contrasts. The observed enhancement of inter-areal communication with increased feedback suggests a key role for top-down feedback, to dynamically modulate the efficiency of information transfer between cortical areas without altering the underlying structure of the communication subspace (see below, Dynamic modulation of functional connectivity through feedback), a finding consistent with previous work on subspace dimensionality [33].

### Frequency decomposition of communication subspaces

**Figure 4:**
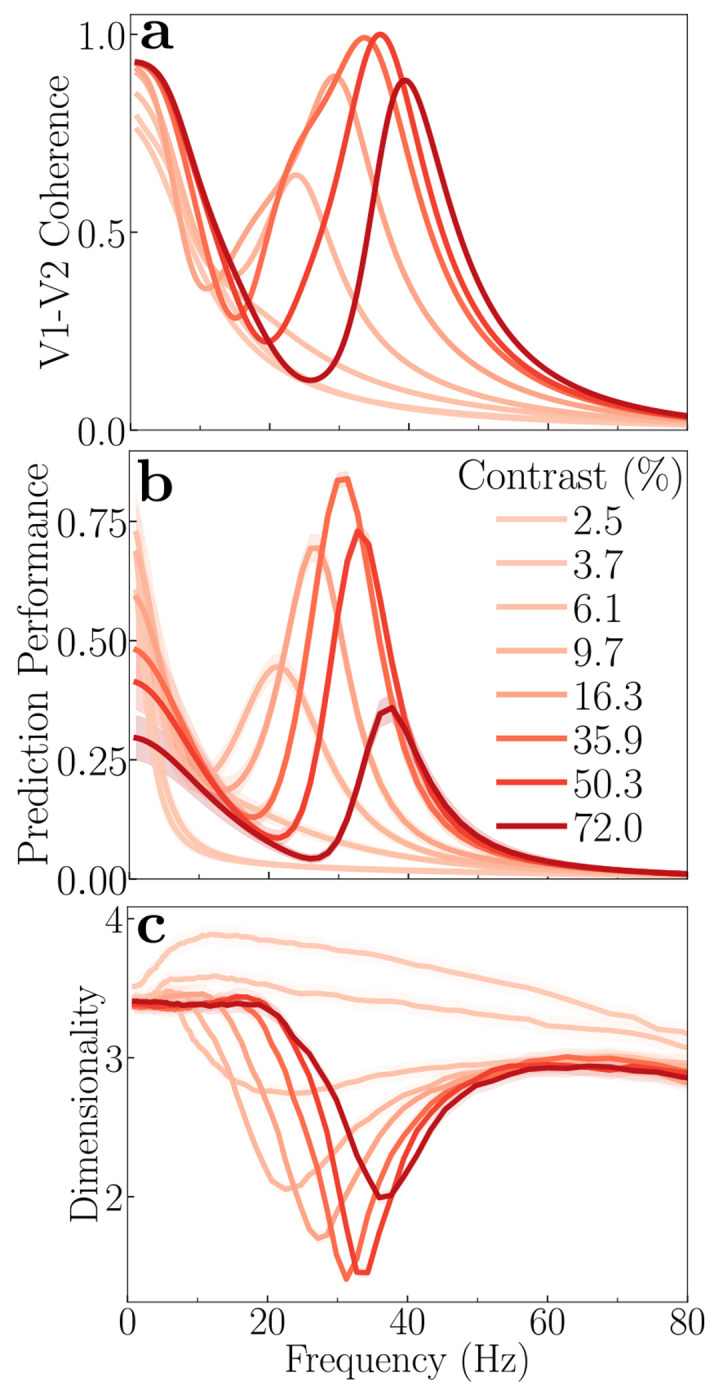
Theoretical prediction: Frequency-dependence of communication. **a,** V1-V2 coherence spectra for different contrasts; same as in Fig. 2e. **b,** Prediction performance versus frequency at different contrasts, averaged across different subsets of the source and target populations. At low stimulus contrast (≈ 10 %), a peak in communication efficacy is observed around 20 Hz. As stimulus contrast increases, the preferred frequency of communication shifts towards higher frequencies (40 Hz). The magnitude of communication reaches a maximum around 40% contrast, before declining at higher contrast levels. Under conditions of very low contrast (< 10%), communication is mostly concentrated at very low frequencies. These frequency-specific trends in communication parallel the V1-V2 coherence patterns shown in panel **a**. **c,** Dimensionality of the communication subspace versus frequency, averaged across different subsets of the source and target populations. A notable dip in dimensionality occurs at frequencies corresponding to peaks in communication (panel **b**) and coherence (panel **a**). The shaded areas in panels **b** and **c** represent the standard error of the mean (SEM).

The theory predicts that the effectiveness of communication between brain areas varies systematically with frequency and contrast (Fig. 4). We quantified the efficacy of communication by analytically calculating the prediction performance for each frequency component (see Methods). To achieve this, we transformed the noise correlations of the source and target populations into power spectra using the Wiener-Khinchin theorem. This conversion enabled us to predict the power of the target population from the source population (using a linear readout) at each frequency, thus providing a measure of communication efficacy (prediction performance) as a function of frequency.

V1-V2 communication is dynamically modulated by stimulus contrast in a frequency-dependent manner. At low stimulus contrasts (e.g, approximately 10%), a distinct peak in communication is observed at ≈ 20 Hz (Fig. 4b). As stimulus contrast increased, this preferred frequency for communication shifts toward higher frequencies (Fig. 4b). Furthermore, the magnitude of this communication peak exhibits a non-monotonic relationship with contrast, reaching its maximum at around 40% contrast before declining at higher contrasts. This particular effect is due to the normalization; removing normalization eliminates the peaks in both coherence and prediction performance (see Suppl. Fig. 11f–g). Under conditions of very low contrast, no distinct peak in communication is observed; instead, communication is mostly concentrated at very low frequencies (<10 Hz). Notably, these observed frequency-specific trends in communication bear a striking resemblance to the V1-V2 coherence patterns (Fig. 4a). This parallelism indicates a relationship between the degree of synchronization of neural activity between brain areas and the effectiveness of communication between these brain regions, a prediction that can be tested experimentally (see Discussion).

The dimensionality of the inter-areal (V1-V2) CS also varies with frequency and stimulus contrast (Fig. 4c). The dimensionality of the communication subspace decreases at frequencies where inter-areal coherence and communication are maximal, for each contrast (Fig. 4c).

The dimensionality of the CS is given by the rank of the covariance matrix between the source (V1) and target (V2) subpopulations (see Methods: Interpretation of the results). The dimensionality of the covariance matrix is influenced by three main factors. First, the dimensionality remains low in the absence of normalization (Suppl. Fig. 11h). However, including normalization specifically reduces dimensionality at coherent frequencies (Fig. 4c). Second, the dimensionality is increased for a fully interconnected (all-to-all) connectivity between V1 and V2 (Suppl. Fig. 11l). Third, delocalized inputs (e.g., noise images or naturalistic images) lead to a higher dimensionality than localized inputs (e.g., gratings) (Suppl. Fig. 11p) as observed in experimental results [32].

### Dynamic modulation of functional connectivity through feedback

Anatomical connections influence neural communication but do not completely determine it. Rather, it has been observed experimentally that functional connectivity between brain areas changes dynamically [106–108].

**Figure 5:**
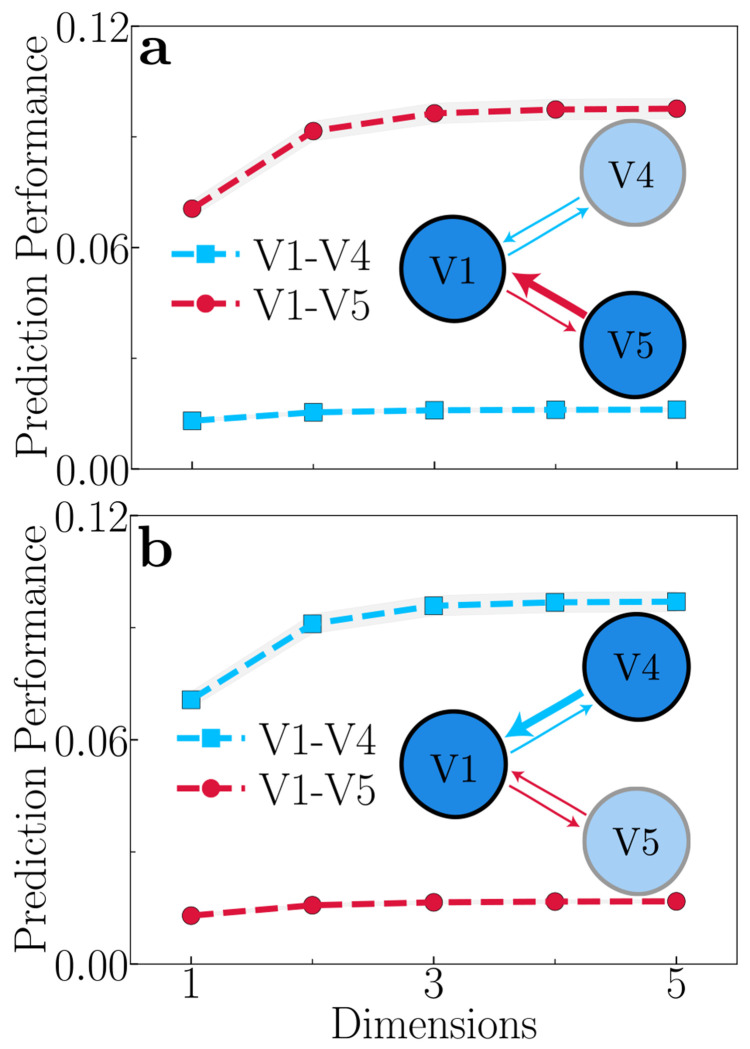
Theoretical prediction: Feedback-dependent modulation of functional connectivity. **a,** Feedback from V5 → V1 is stronger than feedback from V4 → V1, and V1 preferentially enhances communication with V5. **b,** Feedback from V4 → V1 is stronger than feedback from V5 → V1, and V1 preferentially enhances communication with V4. The plotted prediction performance is an average across different subsets of the source and target populations, and the shaded areas represent the standard error of the mean (SEM). These results demonstrate that top-down feedback can dynamically route information flow between cortical areas, i.e., modulating functional connectivity.

Our theory predicts that functional connectivity is controlled by modulating feedback gain (Fig. 5). We expanded our model to encompass three cortical areas: V1, V4, and V5 (also called MT). In this expanded model, V1 has reciprocal connections with both V4 and V5, representing the parallel dorsal and ventral visual pathways, respectively [109–111]. For simplicity, we assume there are no direct connections between V4 and V5 in the model. Analysis of this three-area model provides compelling evidence for the role of top-down feedback in controlling functional connectivity. When the feedback strength from V5 → V1 is greater than the feedback from V4 → V1, we observe that V1 strengthens communication with V5 (Fig. 5a). Conversely, when the feedback from V4 to V1 is stronger, V1 shows enhanced communication with V4 (Fig. 5b). This suggests that the relative strength of feedback from the higher cortical area (V5 or V4) can dictate the information flow from the lower cortical area (V1). One possible way this could be achieved is by selectively enhancing the neural activity in either V5 or V4, which will, in turn, increase the feedback to V1. This is commensurate with experimental evidence that feature-based attention and task demands may selectively boost activity in one or another cortical area [112–118], and specifically that attending to motion increases the transfer of motion information (communication) between V1 and V5 [119–121]. Therefore, top-down signals can dynamically modulate functional connectivity from lower to higher cortical areas, simply by adjusting the gain of neural activity in one higher cortical area (e.g., V4) relative to another (e.g., V5). This mechanism enables flexible cortical information routing, which is essential for cognitive flexibility.

Note that, to isolate the variable of interest, we modeled V4 and V5 with identical parameters for their recurrent, feedforward (V1 → V4/V5) and feedback (V1 ← V4/V5) connectivity. This ensures that the observed increase in communication with either V4 or V5 is a direct consequence of the stronger feedback from that specific area.

### Robustness with respect to model parameters

All the results in this paper are obtained from the same set of parameters (Table 1) unless otherwise specified. To ensure the robustness of our findings, we verified them against a wide range of model parameters.

We systematically repeated the calculations and simulations described above, varying each parameter individually while holding others at their baseline values. These parameter ranges are specified in Table 2. Overall, our key findings remain robust across a wide range of parameter regimes without requiring precise parameter tuning:

- Contrast-response functions (Fig. 2a). Higher cortical area (V2) consistently exhibits greater contrast gain, reflected by a lower semisaturation contrast and a steeper exponent than lower cortical area (V1).
- Gamma oscillations (Fig. 2c,e). Both the power spectra and coherence spectra exhibit prominent gamma-band peaks that shift to higher frequencies as contrast increases.
- Alpha oscillations (Fig. 3c). Low contrast inputs and weak feedback gain produce clear alphaband peaks in the power spectra.
- Communication subspaces (Fig. 2g). The V1-V2 subspace is lower dimensional than the V1-V1 subspace.
- Communication subspaces and feedback gain (Fig. 3k). The V1-V2 communication strength consistently increases with increasing feedback gain.
- Communication subspaces and input gain (Fig. 3l). V1-V1 and V1-V2 communication strength consistently decrease with increasing input gain.
- Frequency-dependence of communication subspaces (Fig. 4). The communication strength peaks at the same frequencies as coherence for each contrast level, and subspace dimensionality is minimized at those frequencies.
- Dynamic functional connectivity (Fig. 5). Enhancing feedback from V4 to V1, selectively boosts communication between V1 and V4, and likewise, enhancing feedback from V5 to V1, selectively enhances communication between V1 and V5.
- Oscillatory activity depends on normalization (Suppl. Fig. 11e,f). Removing normalization abolishes the gamma frequency peaks in both power and coherence spectra.
- Dimensionality of inter-areal communication subspaces (Suppl. Fig. 11l,p). The dimensionality is low when the input drive is localized (e.g., gratings). However, delocalized input drives (e.g., naturalistic images) result in a higher dimensionality. Fully all-to-all connections between V1 and V2 also produce a higher subspace dimensionality.

## Discussion

We introduce an analytically tractable theory of neural circuit function that implements divisive normalization dynamically across multiple interconnected brain areas. Emergent properties of the theory account for a diverse array of neurophysiological phenomena, including oscillatory activity (measurable as peaks in LFP power and coherence spectra) and communication subspaces. Based on the theory, we propose a unified view of inter-areal communication with the following experimentally-testable predictions: i) increasing feedback strength should enhance inter-areal communication and diminish within-area communication, without altering the subspace dimensionality; and ii) frequencies exhibiting high coherence should correspond to both stronger communication and reduced subspace dimensionality. Furthermore, the theory proposes a mechanism for dynamic functional connectivity: top-down feedback signals enhance communication between brain areas simply by adjusting the gain of neural activity in one or more cortical areas. This modulation of neural gain offers a broader mechanism for dynamically changing the functional connections between brain areas over time.

### Neural circuit function

The original ORGaNICs model [16, 17], in its initial form without feedback connections, serves as a biophysically-plausible counterpart to Long Short Term Memory units (LSTMs) [122], a class of artificial recurrent neural networks (RNNs) prevalent in machine-learning applications [123–126]. Unlike LSTMs or any other previously proposed RNNs, ORGaNICs have built-in recurrent normalization, which offers several advantages, including stability in the network even in circuits with exuberant recurrent connections [18, 19].

The hierarchical version of the ORGaNICs theory introduces feedback connections, drawing a partial analogy to “hierarchical predictive coding” models of sensory and perceptual processing [127–132]. However, the hierarchical model diverges from predictive coding in its fundamental mechanisms. In predictive coding, it is proposed that forward-propagating signals represent prediction errors, while feedback pathways convey predictions to lower levels of hierarchy. The goal is to minimize prediction error by matching sensory input with top-down predictions. In hierarchical ORGaNICs, instead of simply propagating prediction errors, the interplay between feedforward and feedback drives adjusts neuronal responses to a value that balances the feedforward processing of its inputs with the feedback (see Ref. [15] for details). This mechanism is more consistent with neurophysiological and psychophysical phenomena than predictive coding models. For instance, neurons maintain sustained activity in response to a predictable stimulus [133], whereas predictive coding theories would suggest that the activity, representing prediction error, should be diminished or “explained away” as the stimulus becomes predictable.

### Normalization

The proposed theory has an analytical fixed point that follows the normalization equation (Eq. 2), for arbitrary (non-negative) normalization weights, when the input drive is constant over time (see Appendix for derivation) [18, 19]. The model achieves normalization dynamically via recurrent amplification, which is inversely regulated by the activity of inhibitory modulator cells. When the input is weak, the modulator cells have a small response, resulting in strong recurrent amplification. Conversely, a strong input generates a large response from modulator cells, which suppresses the amplification. This process modulates both excitatory and inhibitory recurrent signals, which is consistent with the experimental observation that normalization involves a decrease in both recurrent excitatory and inhibitory conductances [134]. Because the theory successfully implements normalization, it is commensurate with a wide range of physiological and psychophysical phenomena previously explained by the normalization model (see Introduction for references). The functional importance of this mechanism is further validated by our model. When divisive normalization is removed from the model, oscillatory peaks in both the power and coherence spectra are eliminated (Suppl. Fig. 11e,f). Also, the relationship between coherence, prediction performance, and subspace dimensionality is lost (Suppl. Fig. 11g,h).

### Role of feedback

This paper provides an experimentally testable computational model of cortical function where neural activity is shaped by a combination of feedforward drive (bottom-up) and feedback drive (top-down). Feedback in the model may serve a variety of functions. First, feedback provides a means for controlling inter-areal communication (functional connectivity) simply by changing the gain of neural activity in one or more cortical areas (see below, Attention and functional connectivity). Second, the feedback drive may contribute to selective attention (see below, Attention and functional connectivity). Third, the relative strength of the input gain and feedback gain determines whether neural responses are driven bottom-up, top-down, or a combination of the two. Fourth, feedback may contribute to learning to minimize prediction error [15, 135]. In addition, feedback to inhibitory neurons is essential for maintaining network stability.

The interplay between the input gain and the feedback gain determines the primary driver of neural responses. If the input gain is strong and the feedback is weak, the system behaves like traditional feedforward models. Conversely, with strong feedback and weak input, the network is similar to generative models [136–138], creating sensory representations from higher-level abstract knowledge, akin to recalling an image from memory (see Ref. [15] for details). When input and feedback are balanced, the framework integrates prior beliefs with sensory evidence, akin to models based on Bayesian inference [139–145]. The model predicts differential effects of changing input gain versus feedback gain on the power, coherence spectra, and inter-areal communication (see Fig. 3). Hence, these metrics may be used as a signature for distinguishing changes in input gain from changes in feedback.

### Unified view of inter-areal communication

Our theoretical framework offers a novel perspective on inter-areal communication. Rather than viewing “communication through coherence” (CTC) and “communication subspace” (CS) as alternative hypotheses, our theory unifies them, showing that coherence and communication subspaces are complementary empirical phenomena emerging naturally from the same underlying mechanism. We found that the frequency decomposition of communication subspaces closely mirrors the patterns of inter-areal coherence at different contrasts. Specifically, frequencies exhibiting high coherence are also those for which the communication efficacy within the subspace is maximal, and the subspace dimensionality is minimized. Consequently, coherence might dynamically shape the communication subspaces, for instance, by reducing their dimensionality at preferred frequencies [34]. Moreover, the predicted frequency-dependence of communication subspaces might serve as a marker for testing neural circuitry theories (such as ours).

### Attention and functional connectivity

The theory provides a possible explanation for the efficient routing of sensory information between cortical areas, i.e., for changing functional connectivity. Lower cortical areas, such as V1, can flexibly relay information to specific higher cortical areas (e.g., V4 vs. V5) based on the relative strength of the feedback from each of the higher cortical areas. The strength of the feedback can be controlled either by changing the feedback gain or (perhaps more simply) by changing the gain of the neural responses in each of the higher cortical areas. We hypothesize that this feedback-mediated process may account for changes in functional connectivity [108, 146, 147] and feature-based attention [112–116]. Consistent with this hypothesis, experimental evidence demonstrates that feature-based attention is spatially global, i.e., that neural activity is boosted in feature-selective neurons with receptive fields covering the visual field [113, 148].

The theory may also provide insights into the neural processes underlying spatially-selective attention. According to the theory, there are two processes by which attention may modulate the gain of neural activity: i) attention may modulate the input gain of the sensory neurons, following earlier work on the normalization model of attention [53]; ii) attention may modulate the activity of the sensory neurons via the feedback drive. One or the other of these two proposed mechanisms for attentional modulation (e.g., changing the input gain or the feedback drive) may offer a better fit to electrophysiology and behavioral data. Alternatively, one of the mechanisms (e.g., changing the input gain) may offer a better explanation of exogenous attention, whereas the other mechanism (e.g., the feedback drive) may offer a better explanation of endogenous attention. Importantly, the theory predicts different effects of changing input gain versus feedback gain on the power and coherence spectra, as noted above, so these metrics may be used as a signature for distinguishing changes in input gain from changes in feedback.

### Thalamo-cortical model

Alpha oscillations are a defining characteristic of thalamo-cortical dynamics, particularly prominent within the visual system during states of “relaxed wakefulness” [149–151]. Specifically, alpha oscillations are hypothesized to be generated and modulated by thalamo-cortical loops [151, 152], suggesting that alpha rhythms signify a state of functional inhibition or “idling” in sensory pathways, potentially gated by corticothalamic feedback [152, 153]. Our theory predicts the emergence of oscillatory activity in the alpha band (8-12 Hz) under conditions of low stimulus contrast and low feedback to excitatory neurons, and high feedback to inhibitory neurons (*γ* < 1, Figs. 3c,g). This aligns well with the existing literature demonstrating the role of alpha oscillations in mediating cortical inhibition [153, 154]. This positions the theory as a potential tool for modeling the thalamocortical interactions underlying alpha waves.

Furthermore, we hypothesize that the input gain modulator responses depend in part on thalamocortical loops, hence positioning the theory as a means for modeling the interplay between bottom-up sensory input and top-down feedback within the thalamocortical circuit, the gating of visual information flow, thalamic control of functional cortical connectivity [155] and thalamocortical loops in other neural systems [156–160].

### Limitations

We assume the feedback to be instantaneous, but there is an associated feedback delay [161, 162]. The delay in receiving feedback signals from higher cortical areas is due to the finite speed of signal conduction, processing, and computation. These delays arise from factors like axonal conduction times and synaptic transmission. It will be interesting to investigate how the synchronization between cortical areas changes as a function of feedback delay time.

The feedforward, feedback, and recurrent weight matrices may comprise positive and negative weights. Hence, we posit additional inhibitory interneurons to invert the sign of the responses, corresponding to negative weights in the synaptic weight matrices. These inhibitory neurons need not be one-to-one with their excitatory inputs. Rather, each may compute a partial weighted sum of their inputs corresponding to the negative terms in the matrix multiplications. Regardless, these inhibitory interneurons will introduce an additional delay.

Most of the computations posited by the theory (see Methods, Eqs. 1a, 1c, 1b, and 1d) are biophysically plausible (see below and Ref. [17]), but there are some elements of computation for which the underlying mechanisms are unclear. For example, the weighted sums may be implemented as a push-pull balanced increase in excitatory and decrease in inhibitory synaptic inputs (or vice versa) that injects current without changing conductance [163] and the gain control may be implemented with a push-push balanced increase in excitatory and inhibitory synaptic inputs that changes conductance without injecting current [163, 164]. However, we do not have a hypothesis for the cellular mechanism underlying the element-by-element product of firing rates $y_{1}^{+}⊙u_{1}^{+2}$ in Eq. 1b.

The intrinsic time constants are unrealistically short for individual neurons (see Table 1). If more realistic time constants were used, the oscillatory activity and coherence in the gamma-frequency band would shift toward a lower frequency. Instead, one might interpret the time constants in the theory as effective time constants of a population of neurons rather than interpreting them literally as the intrinsic time constants (capacitance/conductance) of individual neurons.

### Biology plausibility

We hypothesize a correspondence between the variables in the equations that describe the theory and cortical microcircuits composed of identified cell types. Unlike generic E-I models (e.g., Wilson and Cowan [165]), we propose that different cell types (different morphology and biochemistry) perform different computations, each with a different output nonlinearity (i.e., F-I curve) transforming membrane potentials to firing rates (see Appendix). Specifically, principal cells **y** may correspond to pyramidal cells, inhibitory modulatory cells **a** may correspond to a combination of parvalbumin-expressing (PV+) cells and somatostatin-expressing (SST+) cells, and inhibitory interneurons **q** may correspond to vasoactive intestinal polypeptide (VIP) cells. Principal cells **y** provide excitatory input to both inhibitory modulatory cells **a** (via **u**) and inhibitory interneurons **q**, inhibitory interneurons **q** provide inhibitory input to inhibitory modulatory cells **a**, and inhibitory modulatory cells **a** provide inhibitory input to principal cells **y** (Fig. 1). This arrangement is commensurate with empirical observations of cortical circuits: pyramidal cells provide excitatory input to PV+, SST+, and VIP cells, VIP cells provide inhibitory input to SST+ cells, and both PV+ and SST+ cells provide inhibitory input to pyramidal cells [166–170]. The inhibitory modulator cells **a** are hypothesized to have large RFs and broad orientation-selectivity (reflecting properties of the normalization pool), consistent with the response properties of SST+ and PV+ neurons, respectively [167, 171, 172]. We also hypothesize excitatory feedback from neurons in higher cortical areas to both principal cells **y** and inhibitory modulatory cells **a** (Fig. 1). Feedback to principal cells may be received by the distal apical dendrites of pyramidal cells in layer 1 (see below). Feedback to inhibitory modulator cells is commensurate with experimental evidence that feedback from higher cortical areas targets SST+ neurons [173–175], and that normalization depends in part on loops through higher visual cortical areas [176–179]. A possible discrepancy between the model and the actual cortical circuit is that the excitatory input from principal cells **y** to inhibitory modulatory cells **a** passes through another excitatory cell type **u** whereas pyramidal cells provide direct excitatory input to PV+ and SST+ cells. One could, however, rewrite the equations for the theory (see Methods, Eqs. 1c and 1b) such that principal cells provide direct excitatory input to inhibitory modulatory cells (specifically by substituting the expression for **u** from Eq. 1b into the expression for **a** in Eq. 1c).

**Figure 6:**
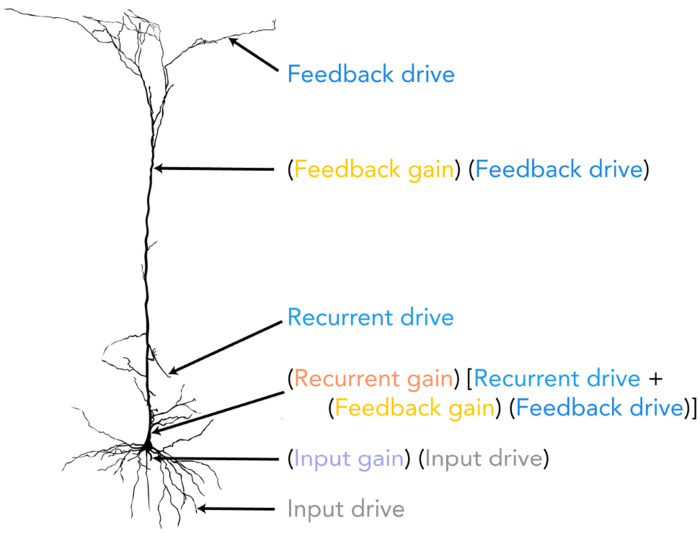
Hypothesized mapping of computational components in the model onto the dendritic compartments of a pyramidal cell. The input drive is hypothesized to be the weighted sum of feedforward inputs arriving at the distal basal dendrites. This is modulated by an input gain, corresponding to inhibition at the proximal basal dendritic trunk. In the apical tuft, the feedback drive represents a weighted sum of feedback inputs from higher cortical areas. This signal is amplified by a feedback gain within the distal apical trunk. The recurrent drive corresponds to the sum of lateral inputs on the proximal apical dendrites. The combined recurrent and feedback signals are then modulated by a recurrent gain at the proximal apical trunk. The overall synaptic current is a combination of these gain-modulated drives. The figure is taken from [185].

We hypothesize a correspondence between the computations performed by the principal cells (**y**) and the computational processes occurring within the dendritic compartments of pyramidal cells. The equation governing the responses of the principal cells (see Fig. 6 and Methods, Eq. 1a) expresses the following synaptic current: (input gain) (input drive) + (recurrent gain) [recurrent drive + (feedback gain) (feedback drive)]. The input drive may be computed as a weighted sum of feedforward inputs in the distal basal dendrites, commensurate with evidence that feedforward synaptic inputs contact basal dendrites. The input gain may correspond to inhibitory neurons that contact the most proximal trunk of the basal dendritic tree. The feedback drive may be computed as a weighted sum of feedback inputs received by distal apical dendrites in layer 1, which are known to receive signals from higher cortical areas. The feedback gain might be attributed to the amplification of the feedback drive within the apical trunk of pyramidal cells [180]. The recurrent drive may correspond to a weighted sum of lateral inputs at more proximal apical dendrites, and the recurrent gain may correspond to inhibitory inputs that contact the most proximal trunk of the apical dendritic tree.

Experimental evidence suggests that acetylcholine (ACh) selectively adjusts the balance between bottom-up sensory inputs and top-down signals related to expectancy and uncertainty [181]. It has also been hypothesized that ACh signals when bottom-up sensory inputs are known to be reliable [182, 183]. Consequently, it is reasonable to hypothesize that the input gain and/or feedback gain might be controlled (at least in part) by ACh. Although ACh is released broadly throughout the cortex, its effect can be regionally specific [184], possibly offering a mechanism for how the values of the state parameters can differ across the hierarchy of brain areas.

## Methods

### The model

We propose a multistage hierarchical recurrent circuit that incorporates feedforward connections from the primary visual cortex (V1) to higher visual cortical areas (e.g., V2, V4, MT) and feedback connections from these higher brain areas to V1 (Fig. 1). We refer to V2 throughout this paper for concreteness, but it is merely an example of a higher brain area that is reciprocally connected with V1. This model builds on the ORGaNICs framework developed by Heeger et al. [16, 17]. Divisive Normalization (DN) is achieved in each brain area by local inhibitory neuronal populations.

The response of principal excitatory neurons in the model is a sum of three key components: 1) input drive, a weighted sum of neural responses from a brain area that provides feedforward input (e.g., LGN → V1 and V1 → V2), modulated by input gain; 2) feedback drive, a weighted sum of neural responses from the higher cortical area (e.g., V1 ← V2 or V1 ← V4), modulated (viz., scaled) by feedback gain; and 3) the sum of recurrent drive—a weighted sum of neuronal responses from other neurons in the same brain area (e.g., V1 → V1 and V2 → V2)—and scaled feedback drive, collectively modulated by recurrent gain.

The proposed neural circuit comprises four distinct cell types, the principal excitatory neurons (**y**), the modulatory excitatory neurons (**u**), the modulatory inhibitory neurons (**a** and **q**), with the following dynamics:

(1a) $$\tau_{y_{1}}\frac{\text{d}y_{1}}{\text{d}t}=-y_{1}+\frac{\beta_{1}}{2}⊙z_{1}\;+\left(\frac{1}{1+a_{1}^{+}}\right)\;\left(W_{11}\sqrt{y_{1}^{+}}+\frac{\gamma_{1}}{2}⊙W_{12}\sqrt{y_{2}^{+}}\right)$$

(1b) $$\tau_{u_{1}}\frac{du_{1}}{dt}=-u_{1}+{\left(\frac{\sigma_{1}}{2}\right)}^{2}+N_{1}\left(y_{1}^{+}⊙u_{1}^{+2}\right)$$

(1c) $$\tau_{a_{1}}\frac{da_{1}}{dt}=-a_{1}+\frac{W_{12}\sqrt{y_{2}^{+}}}{2\cdot q_{1}^{+}}+u_{1}^{+}+a_{1}⊙u_{1}^{+}+\alpha_{1}\frac{du_{1}}{dt}$$

(1d) $$\tau_{q_{1}}\frac{dq_{1}}{dt}=-q_{1}+\sqrt{y_{1}^{+}}.$$

**Figure 7:**
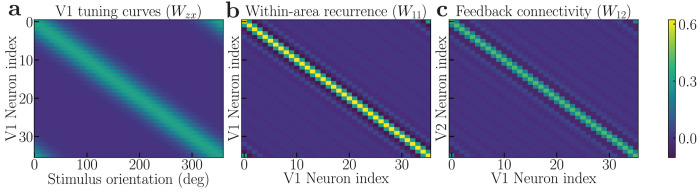
Connectivity matrices in the hierarchical recurrent circuit model. ***a***, *The V1 encoding matrix (***W***_zx_), each row represents the tuning curve of a V1 neuron.* ***b****, The recurrent connectivity matrix for V1 (***W_11_***). The V2 recurrent connectivity matrix* **W**_**22**_ *has a similar structure.* ***c****, The Feedback connectivity matrix from V2 to V1 (***W_12_***). The corresponding feedforward matrix from V1 to V2, (***W_21_***), is its transpose. Since* **W_21_** *is symmetric, the feedforward and feedback matrices are identical (***W_12_** = **W_21_***)*.

The above equations are for V1; V2 follows a similar set of equations (see Appendix). The membrane potentials of neurons in cortical areas V1 and V2 are denoted by **y**_1_ and **y**_2_, respectively, while their corresponding firing rates are represented by $y_{1}^{+}$ and $y_{2}^{+}$. The ⊙ symbol represents the Hadamard product, element-wise multiplication of vectors. To estimate the firing rates $y_{1}^{+}$ and $y_{2}^{+}$ from their respective membrane potentials, we employ a modified Gaussian Rectification (GR) model [186, 187]. The variable **z**_1_ denotes the input drive to V1 from the preceding brain area (the lateral geniculate nucleus of the thalamus, LGN), computed as a weighted ($W_{zx}$) sum of the LGN firing rates. $\tau_{k}$ is the intrinsic time constant for each subtype of neuron, where k=y,u,a,q. $W_{11}\sqrt{y_{1}^{+}}$ and $W_{12}\sqrt{y_{2}^{+}}$ are the recurrent and feedback drive, respectively, where **W**_11_ is the recurrent weight matrix for V1. **W**_11_ has a center-surround structure where the closer connections are excitatory and the more distant connections are inhibitory (Fig. 7b), same as in [17] where the feedforward ORGaNICs was introduced. The recurrent weight matrix for V2, **W**_22_, has a similar structure. The maximum eigenvalue for these recurrent weight matrices is set to unity to ensure stability. However, we have shown that this need not be the case because normalization ensures stability [18, 19]. **W**_12_ represents the interareal feedback connectivity matrix from V2 to V1, its entries are all non-negative, and the matrix is diagonally dominant (Fig. 7c). The feedforward matrix from V1 to V2, **W**_21_, is taken to be the transpose of **W**_12_. Given that **W**_12_ is symmetric, it follows that **W**_12_ = **W**_21_. The normalization matrix in V1 (**N**_1_) and V2 (**N**_2_) is taken to be all ones, meaning each neuron contributes equally to the normalization. The input gain and feedback gain for V1 are modulated by *β*_1_ and *γ*_1_, respectively. The recurrent and feedback amplifications in V1 are modulated dynamically by the inhibitory neuron population **a**_1_.

Both the V1 and V2 neurons follow the normalization equation *exactly* when *β* and *γ* are set to unity (i.e. when there is identical feedback to excitatory (**y**_1_) and inhibitory (**a**_1_) neurons) and **W**_11_ is the identity matrix (i.e., each neuron recurrently amplifies itself), but we have shown that the circuit closely approximates normalization for a wide range of recurrent weight matrices (**W**_11_) [19]. For V1 neurons, normalization is expressed as:

(2) $$y_{1}^{+}=\frac{{\left\lfloor z_{1}\right\rfloor }^{2}}{\sigma^{2}+N_{1}{\left\lfloor z_{1}\right\rfloor }^{2}},$$

and likewise for V2

(3) $$y_{2}^{+}=\frac{{\left\lfloor z_{2}\right\rfloor }^{2}}{\sigma^{2}+N_{2}{\left\lfloor z_{2}\right\rfloor }^{2}},$$

where $z_{2}=W_{21}y_{1}^{+}$ is the drive from V1 to V2, *σ* is the semi-saturation constant. Normalization is achieved dynamically via recurrent amplification (amplifying weak inputs but not strong inputs) with the three modulatory neurons, **u**_1_, **a**_1_ and **q**_1_ whose dynamics are governed by Eq. 1b, Eq. 1c, and Eq. 1d respectively. The equations for modulatory neurons are derived by substituting the normalization equation (Eq. 2) in the equation for the principal neurons (Eq. 1a) (see Appendix for derivation). An increase in the activity of principal neurons (**y**_1_) leads to enhanced activation in **u**_1_ and **q**_1_. This, in turn, drives a net increase of activity in modulatory neurons (**a**_1_). The elevated **a**_1_ activity subsequently exerts an inhibitory effect on **y**_1_, thereby implementing normalization by the recurrent interactions between the neural populations (see Appendix for derivation).

The parameter *γ*_1_ modulates the feedback gain to the principal excitatory neuron population (**y**_1_). This determines the relative feedback to the excitatory neurons (**y**_1_) compared to the inhibitory neurons (**a**_1_). As a rule of thumb, *γ*_1_ < 1 indicates greater feedback inhibition, *γ*_1_ = 1 denotes a balance between feedback excitation and inhibition, and *γ*_1_ > 1 signifies more feedback excitation. For simplicity, we have set the feedback to the inhibitory neurons (**a**_1_) at a fixed value of 0.50 (hence, the factor of $\frac{1}{2}$ in equation Eq. 1c and Eq. 1a, see Appendix for details). Similarly, the parameter *β*_1_ modulates the relative input gain to the principal excitatory neuron population (**y**_1_) compared to the modulatory excitatory neuron population (**u**_1_). For simplicity, we set the input gain to the modulatory neurons (**u**_1_) to 0.50 (hence, the factor of $\frac{1}{2}$ in equation Eq. 1a and Eq. 1b, see Appendix). Note that when *β*_1_ and *γ*_1_ are both set to unity and the recurrent matrix is identity, the network achieves excitatory-inhibitory balance exactly, and the normalization (Eq. 2) is followed precisely.

We modeled each brain area using a ring model, where principal neurons are arranged in a circle to represent continuous features like stimulus orientation. The recurrent connectivity between these neurons is determined by the distance between them (Fig. 7b). Each modeled area consists of 72 neurons for each of the four specified types (**y**_1_,**u**_1_,**a**_1_,**q**_1_). To simulate sensory input, a visual stimulus with a defined contrast and orientation is presented. The stimulus is then convolved with the tuning curves of the V1 neurons to provide the input drive to the V1 principal neurons. The tuning curves are modeled as raised cosine functions, each peaking at the neuron’s preferred orientation (Fig. 7a).

### Power and Coherence Spectra

The time evolution of neural population activity in V1 and V2 can be described by a general stochastic differential equation (SDE) with an additive noise term **L**d**W**:

(4) dx=f(x,u)dt+LdW,

where $x\in {\mathbb{R}}^{n}$ is a vector representing the activity of neurons; $f(x,u)\in {\mathbb{R}}^{n}$ represents the deterministic part of the dynamics explained above (Eq. 1); d**W** is a vector of independent Gaussian increments with correlation matrix $D\left(𝔼\left[dW\cdot dW^{⊤}\right]\right)$, and $L\in {\mathbb{R}}^{n\times n}$ is the dispersion matrix defining how noise enters the system. Given the analytical solution for the fixed point of the dynamical system (Eq. 2), we can linearize the system around the fixed point (x*):

(5) $$dx=J_{x^{*}}\left(x-x^{*}\right)dt+LdW,$$

where $J_{x^{*}}$ is the Jacobian computed at the fixed point $x^{*}$. This linearization leads to a wide-sense stationary Gaussian process, x(t), whose power spectral density matrix, $𝓢(\omega )\in {\mathbb{R}}^{n\times n}$, is given by [188]:

(6) $$𝓢(\omega )=𝔼\;\left[|\overset{ˆ}{x}(\omega ){|}^{2}\right]=(ı\omega I+J{)}^{-1}{LDL}^{⊤}(-ı\omega I+J{)}^{-⊤}.$$

From 𝓢(ω), we can directly compute the coherence, $\kappa_{ij}$, between any two i and j neurons in the two areas:

(7) $$\kappa_{ij}=\frac{{\left|{𝒮}_{ij}\right|}^{2}}{{𝒮}_{ii}{𝒮}_{jj}}.$$

### Communication subspaces

Here we present the method used to characterize the communication subspaces between two subpopulations (source and target) of neurons [32, 162, 189, 190]. These subpopulations may comprise distinct neurons in the same brain area or across different brain areas. The basic idea is to determine how well the fluctuations in responses of a target subpopulation can be predicted using a linear transform (a weighted sum) of the fluctuations in responses of the source subpopulation, and to determine the dimensionality of this linear transform.

The fluctuations in the responses and their covariability are fully determined by the correlation matrix at steady-state when the strength of the noise is small. Therefore the correlation matrix, denoted as **C**(0), for the responses **x**(*t*) can be computed by solving the Lyapunov equation [188]:

(8) $$JC(0)+C(0)J^{⊤}=-{LDL}^{⊤}.$$

Once **C**(0) is obtained, we follow the method proposed by Semedo et al. [32] to construct an analytical model of the communication subspace. To achieve this, we divide the mean-subtracted responses of the entire neuronal population, denoted as **x**, into two subsets: **s** (responses of the source neurons, e.g., in V1) and **t** (responses of the target neurons, e.g., in V2 or V1). We consider a linear model for predicting target responses using the source responses, $\overset{ˆ}{t}=B^{⊤}s$, where the mean squared error (MSE) is given by:

(9) $$\varepsilon =𝔼\left[{\left|t-B^{⊤}s\right|}^{2}\right]=Tr\left(C_{2}+B^{⊤}C_{1}B-2B^{⊤}C_{3}\right),$$

where $C_{1}=𝔼\left[{ss}^{⊤}\right],C_{2}=𝔼\left[{tt}^{⊤}\right]$, and $C_{3}=𝔼\left[{st}^{⊤}\right]$. These matrices can be directly obtained from C(0). To find the optimal readout matrix $B_{\text{opt}}$, we minimize *ε* with respect to **B**, leading to the Ordinary Least Squares solution:

(10) $$B_{opt}=C_{1}^{-1}C_{3},$$

First, we define the covariance of the target activity *predicted* by the optimal model (note the “hats”), which we denote as ${\overset{ˆ}{C}}_{2}$:

(11) $${\hat{C}}_{2}=𝔼\left[\hat{t}{\hat{t}}^{⊤}\right]=B_{\text{opt}}^{⊤}C_{1}B_{\text{opt}}$$

By substituting the expression for $B_{\text{opt}}$, we arrive at a key relationship:

(12) $${\overset{ˆ}{C}}_{2}={\left(C_{1}^{-1}C_{3}\right)}^{⊤}\;C_{1}\left(C_{1}^{-1}C_{3}\right)=C_{3}^{⊤}C_{1}^{-1}C_{3}$$

The principal dimensions of this predicted covariance matrix, ${\overset{ˆ}{C}}_{2}$, define the communication subspace. These are found by computing the eigen-decomposition:

(13) $${\overset{ˆ}{C}}_{2}=V\Lambda V^{⊤}$$

Here, the columns of the orthogonal matrix **V** are the eigenvectors of ${\overset{ˆ}{C}}_{2}$ and Λ is a diagonal matrix of the corresponding eigenvalues, sorted in descending order. The prediction performance for a reduced-rank approximation of dimension *i* is defined as: A rank-*i* approximation, $B_{\text{RRR}}$, is constructed by projecting the optimal solution $B_{\text{opt}}$ onto the subspace spanned by the top *i* eigenvectors of ${\overset{ˆ}{C}}_{2}$.

(14) $$B_{RRR}(i)=B_{\text{opt}}V(i)V^{⊤}(i)=B_{\text{opt}}P_{i}$$

where V(i) is a matrix containing first i columns of V. Here $P_{i}=V(i)V^{⊤}(i)$ is the projection matrix. Note that $P_{i}$ is symmetric ($P_{i}^{⊤}=P_{i}$) and idempotent ($P_{i}^{2}=P_{i}$). We define the prediction performance as:

(15) $$Performance(i)=1-\frac{\varepsilon_{i}}{\varepsilon_{0}},$$

where $\varepsilon_{i}$ is the MSE for the rank-*i* approximation

(16) $$\varepsilon_{i}=Tr\left(C_{2}+B_{RRR}^{⊤}(i)C_{1}B_{RRR}(i)-2B_{RRR}^{⊤}(i)C_{3}\right).$$

$\varepsilon_{0}$ is the MSE for the case where the readout matrix is a null matrix, corresponding to the total variance of the target population $\left(\varepsilon_{0}=Tr\left(C_{2}\right)\right)$.

To simplify the expressions for $\varepsilon_{i}$. We substitute $B_{\text{RRR}}(i)=B_{\text{opt}}P_{i}$ into the expression:

(17) $$\varepsilon_{i}=Tr\left(C_{2}\right)-Tr\left(2P_{i}B_{opt}^{⊤}C_{3}-P_{i}B_{opt}^{⊤}C_{1}B_{opt}P_{i}\right)$$

Now, we substitute $B_{\text{opt}}^{⊤}=C_{3}^{⊤}C_{1}^{-1}$ and recognize that the term $B_{\text{opt}}^{⊤}C_{1}B_{\text{opt}}$ is ${\overset{ˆ}{C}}_{2}$. Using the cyclic property of the trace (Tr(AB)=Tr(BA)) and the idempotent property of the projection matrix ($P_{i}^{2}=P_{i}$), we can simplify further:

(18) $$\varepsilon_{i}=Tr\left(C_{2}\right)-Tr\left({\overset{ˆ}{C}}_{2}P_{i}\right)$$

By applying the cyclic property and expanding $P_{i}$, we can see that the second term is the sum of the top i eigenvalues $\left(\lambda_{j}\right)$ of ${\overset{ˆ}{C}}_{2}$.

(19) $$Tr\left({\overset{ˆ}{C}}_{2}V(i)V(i{)}^{⊤}\right)=Tr\left(V(i{)}^{⊤}{\overset{ˆ}{C}}_{2}V(i)\right)=\sum_{j=1}^{i}\lambda_{j}$$

Now the expression for prediction performance simplifies to:

(20) $$Performance(i)=\frac{\sum_{j=1}^{i}\lambda_{j}}{Tr\left(C_{2}\right)},$$

Which can also be interpreted as:

(21) $$Performance(i)=\frac{\text{Explained variance}(i\;\text{dimensions})}{\text{Total target variance}}.$$

The maximum prediction performance (full-rank approximation) is given by:

(22) $$\text{Performance}=\frac{Tr\left({\overset{ˆ}{C}}_{2}\right)}{Tr\left(C_{2}\right)}=\frac{Tr\left(C_{3}^{⊤}C_{1}^{-1}C_{3}\right)}{Tr\left(C_{2}\right)}$$

#### Interpretation of the results:

The prediction performance (Eq. 22) is governed by the interplay between the source (**C**_1_), target (**C**_2_), and cross-population (**C**_3_) covariances. The effectiveness of communication (indicated by a large predictive performance) can be understood through three key factors: 1) *Source-target correlation*: Prediction performance is directly enhanced by a stronger correlation between the source and target populations (i.e., larger **C**_3_ norm). 2) *Signal reliability*: Prediction performance is boosted by the reliability of the signals. Less noisy activity in source and target populations, indicated by smaller diagonal entries in **C**_1_ and **C**_2_, leads to better prediction performance. 3) *Alignment*: Prediction performance is maximized when the principal directions of source covariance (eigenvectors of **C**_1_) align with the principal directions of source-target covariance (left singular vectors of **C**_3_).

This framework also explains the results in the frequency domain. The key metric here is the cross-power spectrum, which is the Fourier transform of the cross-covariances, and coherence, which is the squared cross-power spectrum normalized by the product of the auto-spectra, Eq. 7. Frequencies that show high coherence, indicating high cross-covariances between source and target, the prediction performance is highest at these specific frequencies. Note that the dimensionality of the communication subspace is given by the rank of the matrix ${\overset{ˆ}{C}}_{2}=C_{3}^{⊤}C_{1}^{-1}C_{3}$. Since the source covariance matrix **C**_1_ is a full-rank matrix, the dimensionality of the subspace is determined exactly by the rank of the cross-covariance matrix, **C**_3_. In the frequency domain, this means the dimensionality at a specific frequency is given by the rank of the cross-power spectral density matrix. Our results show that the extent of normalization from surrounding neurons plays a critical role in shaping the dimensionality of the communication subspace. With surround normalization, dimensionality decreases specifically at frequencies where coherence is strongest (Fig. 4c). In contrast, without this normalization, no such correlation between coherence and dimensionality is observed (Fig. 11h).

### Frequency decomposition of the communication subspace

To analyze how communication between different brain areas varies across frequencies, we consider the frequency decomposition of the neural responses. The Wiener-Khinchin theorem states that the autocorrelation function C(τ) is the inverse Fourier transform of the power spectral density (PSD) matrix (𝓢(ω)):

(23) $$C(\tau )=\int_{-\infty }^{\infty }𝓢(\omega )e^{ı\omega \tau }d\omega$$

To isolate C(τ) at a particular frequency ($\omega^{*}$), we apply an idealized narrow band-pass filter centered at $\omega^{*}$. Specifically, the filter’s transfer function H(ω) is given by,

(24) $$H(\omega )=\left\{\begin{array}{ll} 1 & \text{if}\;\omega \in A\\ 0 & \text{otherwise} \end{array}\right.$$

where $A=\left[\omega^{*}-\epsilon ,\omega^{*}+\epsilon \right]\cup \left[-\omega^{*}-\epsilon ,-\omega^{*}+\epsilon \right]$ and *ϵ* is a small positive value defining the narrow bandwidth 2*ϵ* around $\omega^{*}$. The spectral density matrix of this filtered signal is given by,

(25) $${𝓢}_{\mathrm{filtered}}(\omega )=|H(\omega ){|}^{2}𝓢(\omega )$$

The resulting autocorrelation of the filtered signal is expressed as:

(26) $$C_{filtered}(\tau )=\int_{-\infty }^{\infty }{𝓢}_{filtered}(\omega )e^{ı\omega \tau }d\omega \;=\int_{-\infty }^{\infty }|H(\omega ){|}^{2}𝓢(\omega )e^{ı\omega \tau }d\omega .$$

Using the definition of H(ω) and assuming ϵ to be sufficiently small such that 𝓢(ω) and $e^{ı\omega \tau }$ are approximately constant over the integration intervals of width 2ϵ, the frequency-specific autocorrelation at $\omega^{*}$ is:

(27) $${\left.C(\tau )\right|}_{\omega^{*}}\approx 2\epsilon \left(𝓢\left(-\omega^{*}\right)e^{-ı\omega^{*}\tau }+𝓢\left(\omega^{*}\right)e^{ı\omega^{*}\tau }\right).$$

At zero time lag (τ=0),

(28) $${\left.C(0)\right|}_{\omega^{*}}\approx 2\epsilon \left(𝓢\left(-\omega^{*}\right)+𝓢\left(\omega^{*}\right)\right).$$

Since the PSD is Hermitian, i.e., $𝓢(\omega )={𝓢}^{*}(-\omega )$), we have

(29) $${\left.C(0)\right|}_{\omega^{*}}=4\epsilon \;Re\left\{𝓢\left(\omega^{*}\right)\right\}$$

This frequency-specific autocorrelation matrix ($\left(C(0)|\omega^{*}\right)$ can then be used in place of C(0) in the communication subspace analysis described above to evaluate the prediction performance at any frequency. As our analysis does not depend on the absolute scaling of this matrix, the prefactor 4ϵ can be disregarded, and we effectively use $Re\left\{𝓢\left(\omega^{*}\right)\right\}$ as the frequency-specific correlation matrix.

## Supplementary Material

1

## Figures and Tables

**Table 1:** Default values of the parameters

*τ*_y_1_,u_1_,a_1_,q_1__	*τ*_y_2_,u_2_,a_2_,q_2__	*γ*_1_	*β*_{1,2}_	*α*_{1,2}_	*σ*_{1,2}_
1 ms	1 ms	1.0	1.0	10	0.07

**Table 2:** Valid parameter ranges

Parameter	Tested stability range
*γ*_1_	[0.0 - 1.1]
*β*_{1,2}_	[0.0 - 2.5]
*α*_{1,2}_	[7.5 - 50]
*σ*_{1,2}_	[0.035 - 0.14]

## Highlights

- Hierarchical neural circuit with reciprocal connections implementing divisive normalization.
- Unified theory of inter-areal communication.
- Emergent properties of the theory explain key experimental findings.
- Feedback from higher cortical areas dictates functional connectivity.

## Appendix Derivation of the General Hierarchical Recurrent Normalization Circuit

This appendix provides a detailed derivation of the general dynamical equations for the hierarchical recurrent normalization model with reciprocal connections. We begin with the primary visual cortex (V1). Assuming **N_1_** is the normalization weight matrix for V1 and **z_1_** is the input drive to V1, we can write the normalization equations for the V1 principal neurons with complementary receptive fields as:

(30)
$$y_{1}^{s+}=\frac{{\left\lfloor z_{1}\right\rfloor }^{n}}{\sigma^{n}+N_{1}\left({\left\lfloor z_{1}\right\rfloor }^{n}+{\left\lfloor -z_{1}\right\rfloor }^{n}\right)}\;\;\;\;\;\;\;y_{1}^{s-}=\frac{{\left\lfloor -z_{1}\right\rfloor }^{n}}{\sigma^{n}+N_{1}\left({\left\lfloor z_{1}\right\rfloor }^{n}+{\left\lfloor -z_{1}\right\rfloor }^{n}\right)}$$

Where $y_{1}^{s+}$ and $y_{1}^{s-}$ represent the steady-state firing rates of principal neurons corresponding to positive $\left({\left\lfloor z_{1}\right\rfloor }^{n}\right)$ and negative ${\left\lfloor -z_{1}\right\rfloor }^{n}$ input drives, respectively. Here, σ represents the semi-saturation constant. The exponent n is typically observed to be n≈2 for the primary visual cortex, it can be different for different brain areas. Mathematically:

(31)
$${\left\lfloor z_{1}\right\rfloor }^{n}+{\left\lfloor -z_{1}\right\rfloor }^{n}={\left|z_{1}\right|}^{n}$$

Our goal is to derive the dynamical equations for a general *n*, that yield the fixed points defined by Eq. 30. First, we distinguish between the membrane potentials and the firing rates of neurons. The membrane potential of a given type of neuron is denoted by vectors such as **y**_1_, **u**_1_, **a**_1_, and **q**_1_. Their corresponding firing rates are $y_{1}^{+}$ (and $y_{1}^{-}$ for complementary responses), $u_{1}^{+},a_{1}^{+}$, and $q_{1}^{+}$. The instantaneous firing rates are related to membrane potentials by applying rectification (denoted by ⌊⋅⌋) followed by power-law activation [191],

(32)
$$x^{+}=\lfloor x\rfloor^{\beta }.$$

The values of *β* are taken to be $\left\{2,\frac{1}{2},1,1\right\}$ for $\left\{y_{1},u_{1},a_{1},q_{1}\right\}$, respectively. Combining the firing rates of principal neurons with the complementary receptive fields, $y_{1}^{s+}$ and $y_{1}^{s-}$, Eq. 30 can be alternatively written as,

(33)
$${\left|y_{1}\right|}^{n}=y_{1}^{s+}+y_{1}^{s-}=\frac{{\left|z_{1}\right|}^{n}}{\sigma^{n}+N_{1}{\left|z_{1}\right|}^{n}}$$

To proceed with our derivation, we need to isolate the ${\left|z_{1}\right|}^{n}$ term. From Eq. 33, we have:

(34)
$${\left|z_{1}\right|}^{n}=\sigma^{n}⊙{\left|y_{1}\right|}^{n}+{\left|y_{1}\right|}^{n}⊙\left(N_{1}{\left|z_{1}\right|}^{n}\right)$$

where ⊙ denotes element-wise multiplication. Using the identity $x_{1}⊙x_{2}=D\left(x_{1}\right)x_{2}$, where $D\left(x_{1}\right)$ is a diagonal matrix with the elements of vector $x_{1}$ on its diagonal, we can rewrite and solve for ${\left|z_{1}\right|}^{n}$:

(35)
$${\left|z_{1}\right|}^{n}=\sigma^{n}⊙{\left|y_{1}\right|}^{n}+D\left({\left|y_{1}\right|}^{n}\right)N_{1}{\left|z_{1}\right|}^{n}$$

Factoring out ${\left|z_{1}\right|}^{n}$:

(36)
$$\left[I-D\left({\left|y_{1}\right|}^{n}\right)N_{1}\right]\;{\left|z_{1}\right|}^{n}=\sigma^{n}⊙{\left|y_{1}\right|}^{n}$$

Multiplying both sides with [**I** – D(|**y**_1_|^*n*^)**N**_1_]^−**1**^ from the left yields:

(37)
$${\left|z_{1}\right|}^{n}={\left[I-D\left({\left|y_{1}\right|}^{n}\right)N_{1}\right]}^{-1}\;\left(\sigma^{n}⊙{\left|y_{1}\right|}^{n}\right)$$

This expression for ${\left|z_{1}\right|}^{n}$ will be used in subsequent steps. Now, we introduce the dynamical equation for the membrane potential **y**_1_ of the principal V1 neurons, consistent with biological computation in pyramidal cells (see Section ‘Biology plausibility’):

(38)
$$\tau_{y_{1}}\frac{dy_{1}}{dt}=-y_{1}+b_{1}⊙z_{1}+\frac{1}{1+a_{1}^{+}}⊙\left(W_{11}\left({\left(y_{1}^{+}\right)}^{\frac{1}{n}}-{\left(y_{1}^{-}\right)}^{\frac{1}{n}}\right)+\gamma_{1}⊙W_{12}{\left(y_{2}^{+}\right)}^{\frac{1}{n}}\right).$$

Here, $\tau_{y_{1}}$ is the time constant, $b_{1}$ is the input gain, $a_{1}^{+}$ represents firing rates of the inhibitory neuron population. $W_{11}$ is the recurrent V1 connectivity matrix, $W_{12}$ is the connectivity matrix containing the synaptic weights from V2 to V1. $W_{12}{\left(y_{2}^{+}\right)}^{\frac{1}{n}}$ represents the feedback signal from V2 to V1, calculated as the weighted sum of the activities of V2 neurons. $\gamma_{1}$ is the feedback gain modulation parameter. $y_{2}^{+}$ is the firing rate of V2 principal neurons, defined as ${\left\lfloor y_{2}\right\rfloor }^{n}$.

For simplicity, we assume that the recurrent weight matrix $W_{11}=I$. Given that ${\left(y_{1}^{+}\right)}^{1/p}-{\left(y_{1}^{-}\right)}^{1/p}=\left\lfloor y_{1}\right\rfloor -\left\lfloor -y_{1}\right\rfloor =y_{1}$ and ${\left(y_{2}^{+}\right)}^{1/n}=\left\lfloor y_{2}\right\rfloor$, the equation for $y_{1}$ simplifies to:

(39)
$$\tau_{y_{1}}\frac{dy_{1}}{dt}=-y_{1}+b_{1}⊙z_{1}+\frac{1}{1+a_{1}^{+}}⊙\left(y_{1}+\gamma_{1}⊙W_{12}\left\lfloor y_{2}\right\rfloor \right)$$

Now at steady state, $\frac{dy_{1}}{dt}=0$,

(40)
$$0=-y_{1}+b_{1}⊙z_{1}+\frac{1}{1+a_{1}^{+}}⊙\left(y_{1}+\gamma_{1}⊙W_{12}\left\lfloor y_{2}\right\rfloor \right)$$

Expanding the terms:

(41)
$$0=-y_{1}+b_{1}⊙z_{1}+\frac{1}{1+a_{1}^{+}}⊙y_{1}+\frac{1}{1+a_{1}^{+}}⊙\left(\gamma_{1}⊙W_{12}\left\lfloor y_{2}\right\rfloor \right)$$

Rearranging terms:

(42)
$$\frac{a_{1}^{+}}{1+a_{1}^{+}}⊙y_{1}=b_{1}⊙z_{1}+\frac{1}{1+a_{1}^{+}}⊙\left(\gamma_{1}⊙W_{12}\left\lfloor y_{2}\right\rfloor \right)$$

Moving the feedback term to the left-hand side:

(43)
$$\frac{a_{1}^{+}}{1+a_{1}^{+}}⊙y_{1}-\frac{1}{1+a_{1}^{+}}⊙\left(\gamma_{1}⊙W_{12}\left\lfloor y_{2}\right\rfloor \right)=b_{1}⊙z_{1}$$

Factoring out **y**_1_:

(44)
$$y_{1}⊙\left(\frac{a_{1}^{+}}{1+a_{1}^{+}}-\frac{1}{1+a_{1}^{+}}⊙\frac{\gamma_{1}⊙W_{12}\left\lfloor y_{2}\right\rfloor }{y_{1}}\right)=b_{1}⊙z_{1}$$

We define a modulatory term $u_{1}^{+}$, such that:

(45)
$$y_{1}⊙u_{1}^{+}=b_{1}⊙z_{1}$$

where:

(46)
$$u_{1}^{+}=\frac{a_{1}^{+}}{1+a_{1}^{+}}-\frac{1}{1+a_{1}^{+}}⊙\frac{\gamma_{1}⊙W_{12}\left\lfloor y_{2}\right\rfloor }{y_{1}}$$

Next, we derive the dynamical equation for **a**_1_, from the definition of $u_{1}^{+}$:

(47)
$$u_{1}^{+}⊙\left(1+a_{1}^{+}\right)=a_{1}^{+}-\frac{\gamma_{1}⊙W_{12}\left\lfloor y_{2}\right\rfloor }{y_{1}}$$

Upon rearranging,

(48)
$$0=-a_{1}^{+}+\frac{\gamma_{1}⊙W_{12}\left\lfloor y_{2}\right\rfloor }{y_{1}}+\left(1+a_{1}^{+}\right)⊙u_{1}^{+}$$

This equation describes the steady state of **a**_1_. It exhibits a form analogous to that of **y**_1_, comprising an input drive and a recurrent drive term. Therefore, we propose the following dynamical equation for the membrane potential **a_1_**:

(49)
$$\tau_{a_{1}}\frac{da_{1}}{dt}=-a_{1}+\frac{\gamma_{1}⊙W_{12}\left\lfloor y_{2}\right\rfloor }{y_{1}}+\left(1+a_{1}^{+}\right)⊙u_{1}^{+}$$

According to Dale’s Law, excitatory neurons, such as **y**_1_, cannot directly inhibit other neurons. Therefore, the division by **y**_1_ implies the existence of an intermediate inhibitory interneuron. We introduce an inhibitory interneuron population **q**_1_, that tracks **y**_1_:

(50)
$$\tau_{q_{1}}\frac{dq_{1}}{dt}=-q_{1}+{\left(y_{1}^{+}\right)}^{1/n}$$

With this interneuron, the dynamic equation for **a**_1_ becomes:

(51)
$$\tau_{a_{1}}\frac{da_{1}}{dt}=-a_{1}+\frac{\gamma_{1}⊙W_{12}{\left(y_{2}^{+}\right)}^{1/n}}{q_{1}^{+}}+\left(1+a_{1}^{+}\right)⊙u_{1}^{+}$$

Now, we return to Eq. 45 to derive the dynamical equation for the modulatory neuron **u**_1_. Starting with $y_{1}⊙u_{1}^{+}=b_{1}⊙z_{1}$, we take the modulus on both sides and raise to the power of n:

(52)
$${\left|y_{1}\right|}^{n}⊙{\left(u_{1}^{+}\right)}^{n}=b_{1}^{n}⊙{\left|z_{1}\right|}^{n}$$

Substituting the expression for ${\left|z_{1}\right|}^{n}$ from Eq. 37:

(53)
$${\left|y_{1}\right|}^{n}⊙{\left(u_{1}^{+}\right)}^{n}=b_{1}^{n}⊙{\left[I-D\left({\left|y_{1}\right|}^{n}\right)N_{1}\right]}^{-1}\left(\sigma^{n}⊙{\left|y_{1}\right|}^{n}\right)$$

We assume that $b_{1}$ has equal components, i.e., $b_{1}=b_{1}1$. Multiplying both sides by $\left[I-D\left({\left|y_{1}\right|}^{n}\right)N_{1}\right]$ and rearranging:

(54)
$$\left[I-D\left({\left|y_{1}\right|}^{n}\right)N_{1}\right]\;\left(\frac{{\left(u_{1}^{+}\right)}^{n}⊙{\left|y_{1}\right|}^{n}}{b_{1}^{n}}\right)=\sigma^{n}⊙{\left|y_{1}\right|}^{n}$$

Multiplying both sides by $D\left(\frac{1}{{\left|y_{1}\right|}^{n}}\right)$:

(55)
$$D\left(\frac{1}{{\left|y_{1}\right|}^{n}}\right)\;\left[I-D\left({\left|y_{1}\right|}^{n}\right)N_{1}\right]\;\left(\frac{{\left(u_{1}^{+}\right)}^{n}⊙{\left|y_{1}\right|}^{n}}{b_{1}^{n}}\right)=D\left(\frac{1}{{\left|y_{1}\right|}^{n}}\right)\;\left(\sigma^{n}⊙{\left|y_{1}\right|}^{n}\right)$$

This simplifies to:

(56)
$$\frac{{\left(u_{1}^{+}\right)}^{n}}{b_{1}^{n}}-N_{1}\left(\frac{{\left(u_{1}^{+}\right)}^{n}⊙{\left|y_{1}\right|}^{n}}{b_{1}^{n}}\right)=\sigma^{n}$$

Since we assumed that **b**_1_ has equal components, we get:

(57)
$${\left(u_{1}^{+}\right)}^{n}-N_{1}\left({\left(u_{1}^{+}\right)}^{n}⊙{\left|y_{1}\right|}^{n}\right)=\sigma^{n}⊙b_{1}^{n}$$

Elementwise multiplying both sides by $u_{1}/{\left(u_{1}^{+}\right)}^{n}$ from left and rearrange:

(58)
$$0=-u_{1}+\frac{u_{1}}{{\left(u_{1}^{+}\right)}^{n}}⊙\left(b_{1}^{n}⊙\sigma^{n}+N_{1}\left({\left(u_{1}^{+}\right)}^{n}⊙{\left|y_{1}\right|}^{n}\right)\right)$$

This equation describes the steady state of **u**_1_. We propose the following dynamical equation for **u**_1_,

(59)
$$\tau_{u_{1}}\frac{du_{1}}{dt}=-u_{1}+\frac{u_{1}}{{\left(u_{1}^{+}\right)}^{n}}⊙\left(b_{1}^{n}⊙\sigma^{n}+N_{1}\left({\left(u_{1}^{+}\right)}^{n}⊙{\left|y_{1}\right|}^{n}\right)\right)$$

These equations (Eq. 39, Eq. 59, Eq. 51, Eq. 50) describe the dynamics of principal excitatory neurons, modulatory inhibitory neurons, and modulatory excitatory neurons in V1. They exhibit the fixed point solution given by the normalization by design. These equations are general for any choice of n and β. We get a particularly simplified form when we set n=2 and $\beta =\left\{2,\frac{1}{2},1,1\right\}$ for $\left\{y_{1},u_{1},a_{1},q_{1}\right\}$, respectively.

Finally we rewrite the set of four dynamical equations for V1 as:

(60)
$$\tau_{y_{1}}\frac{dy_{1}}{dt}=-y_{1}+b_{1}^{\left(y_{1}\right)}⊙z_{1}+\left(\frac{1}{1+a_{1}^{+}}\right)\;\left(W_{11}\sqrt{y_{1}^{+}}+\gamma_{1}^{\left(y_{1}\right)}⊙W_{12}\sqrt{y_{2}^{+}}\right)$$

(61)
$$\tau_{u_{1}}\frac{du_{1}}{dt}=-u_{1}+\left({\left(b_{1}^{\left(u_{1}\right)}\right)}^{2}⊙\sigma^{2}\right)+N_{1}\left(y_{1}^{+}⊙{\left(u_{1}^{+}\right)}^{2}\right)$$

(62)
$$\tau_{a_{1}}\frac{da_{1}}{dt}=-a_{1}+\frac{\gamma_{1}^{\left(a_{1}\right)}⊙W_{12}\sqrt{y_{2}^{+}}}{q_{1}^{+}}+u_{1}^{+}+a_{1}^{+}⊙u_{1}^{+}+\alpha_{1}\frac{du_{1}}{dt}$$

(63)
$$\tau_{q_{1}}\frac{dq_{1}}{dt}=-q_{1}+\sqrt{y_{1}^{+}}$$

where we have decoupled the input gain $b_{1}$ and feedback gain $\gamma_{1}$ across each equation. When $b_{1}^{\left(y_{1}\right)}=b_{1}^{\left(u_{1}\right)}$ and $\gamma_{1}^{\left(y_{1}\right)}=\gamma_{1}^{\left(a_{1}\right)}$, and $W_{11}=I$ we exactly recover the normalization equation, Eq. 2, at steady state for $y_{1}$. Then to reduce the number of parameters we re-define the input gain as $\beta_{1}\equiv b_{1}^{\left(y_{1}\right)}/b_{1}^{\left(u_{1}\right)}$ and fix $b_{1}^{\left(u_{1}\right)}=1/2$. Similarly, we re-define the feedback gain as $\gamma_{1}\equiv \gamma_{1}^{\left(y_{1}\right)}/\gamma_{1}^{\left(a_{1}\right)}$ and fix $\gamma_{1}^{\left(a_{1}\right)}=1/2$. We thus arrive at Eq. 1 of the main text. Finally, note that the term $\alpha_{1}\frac{du_{1}}{dt}$ is added to the dynamics of $a_{1}$ to achieve correct oscillatory behavior. This term does not impact the steady state solution but influences the dynamics.

The dynamical equations for V2 can be derived following a similar procedure:

(64)
$$\tau_{y_{2}}\frac{dy_{2}}{dt}=-y_{2}+b_{2}^{\left(y_{2}\right)}⊙z_{2}+\left(\frac{1}{1+a_{2}^{+}}\right)\;\left(W_{22}\sqrt{y_{2}^{+}}\right)$$

(65)
$$\tau_{u_{2}}\frac{du_{2}}{dt}=-u_{2}+\left({\left(b_{2}^{\left(u_{2}\right)}\right)}^{2}⊙\sigma^{2}\right)+N_{2}\left(y_{2}^{+}⊙{\left(u_{2}^{+}\right)}^{2}\right)$$

(66)
$$\tau_{a_{2}}\frac{da_{2}}{dt}=-a_{2}+u_{2}^{+}+a_{2}^{+}⊙u_{2}^{+}+\alpha_{2}\frac{du_{2}}{dt}$$

(67)
$$\tau_{q_{2}}\frac{dq_{2}}{dt}=-q_{2}+\sqrt{y_{2}^{+}}$$

where, $z_{2}=W_{21}y_{1}^{+}$; and, like for $y_{1}$, we have decoupled the input gain $b_{2}$ across each equation. When $b_{2}^{\left(y_{2}\right)}=b_{2}^{\left(u_{2}\right)}$ and $W_{22}=I$ we exactly recover the normalization equation, Eq. 2, at steady state for $y_{2}$. To reduce the number of parameters, we redefine the input gain as $\beta_{2}\equiv b_{2}^{\left(y_{2}\right)}/b_{2}^{\left(u_{2}\right)}$ and fix $b_{2}^{\left(u_{2}\right)}=b_{2}^{\left(y_{2}\right)}=1/2$. Note that in principle $y_{2}$ can receive feedback from higher cortical areas, in which case the equations would look identical to Eq. 1 but the subscripts change as 1→2 and 2→3.

In addition to the membrane potential, the corresponding firing rates of V1 and V2 neurons are also simulated. These are modeled as low-pass filtered versions of their target rectified power-law transformations. For V1, as an example:

(68)
$$\tau_{y_{1}^{+}}\frac{dy_{1}^{+}}{dt}=-y_{1}^{+}+{\left\lfloor y_{1}\right\rfloor }^{2}$$

(69)
$$\tau_{u_{1}^{+}}\frac{du_{1}^{+}}{dt}=-u_{1}^{+}+\sqrt{\left\lfloor u_{1}\right\rfloor }$$

(70)
$$\tau_{a_{1}^{+}}\frac{da_{1}^{+}}{dt}=-a_{1}^{+}+\left\lfloor a_{1}\right\rfloor$$

(71)
$$\tau_{q_{1}^{+}}\frac{dq_{1}^{+}}{dt}=-q_{1}^{+}+\left\lfloor q_{1}\right\rfloor$$

### Intrinsic neural circuit noise

The influence of noise on the deterministic neural dynamics is treated analytically in this model. We consider two primary sources of noise: firing rate noise originating from the spiking process and synaptic noise.

**i)** Firing rate noise is added to the firing rate vector $k^{+}$, where $k^{+}$ represents various neural population firing rates (e.g., excitatory like $y_{1}^{+},u_{1}^{+}$ or inhibitory like $a_{1}^{+},q_{1}^{+}$). This noise is attributed to the spiking mechanism, whose strength is found by the modified Gaussian Rectification (GR) model [186, 187]. Due to the high degree of redundancy in neural circuits, the strength of this noise is quenched due to the averaging of these redundant signals. The result is that the noise has a negligible impact on the firing rate dynamics [192, 193]. For a given firing rate vector $k^{+}$, with an associated activation exponent β, the noise dynamics are described by:

(72)
$$\tau_{k^{+}}\frac{dk^{+}}{dt}=-k^{+}+\lfloor k\rfloor^{\beta }+\sigma_{k}\cdot \eta (t),$$

where $\tau_{k^{+}}$ is the time constant for the firing rate dynamics of population $k^{+},\sigma_{k}$ represents the noise strengths for different neuron types, and η(t) is a vector of independent Gaussian white noise process, each with zero mean and unit variance.

**ii)** Synaptic noise is modeled as exponentially low-pass filtered Gaussian white noise (**f**), given by the following SDE:

(73)
$$\tau_{f}\frac{df}{dt}=-f+\sigma_{f}\cdot \eta (t),$$

where $\tau_{f}$ is the noise filtering time constant ($\tau_{f}=1\;msec$, approximating the time constant of synaptic transmission), and $\sigma_{f}$ represents the noise strength. This filtered noise (**f**) is then added to the dynamical equations for the membrane potentials of each neuron, Eq. 60–67, and it is the main source of intrinsic noise.

Upon incorporating these sources of noise, we get a high-dimensional nonlinear stochastic dynamical system with the following variables:

- Variables representing the membrane potential for different neuron types implementing normalization (Eq. 60–67).
- Firing rates (Eq. 72) for each membrane potential variable.
- Filtered synaptic noise (Eq. 73) for each membrane potential variable.

We then linearize this dynamical system about the fixed point found by simulating the deterministic part of the dynamical system, which gives us the Jacobian matrix **J**. We also construct the dispersion matrix **L** and the diffusion matrix **D** using the strength of the noise added in various dynamical variables. The definition of these matrices allows us to compute the covariance matrix and power spectra analytically as discussed in the Methods section of the main text.

### Extrinsic noise in the local field potential

In addition to the noise sources detailed above, we also account for additional sources of noise that contribute to LFP power and coherence spectra. We consider two components of extrinsic noise: **i)** Correlated noise ($\zeta_{\text{correlated}}$), assumed to stem from the volume conduction of electric fields generated by transmembrane current through the extracellular brain tissue, with negligible impact on spiking activity and firing rates [97–99]. **ii)** Uncorrelated noise ($\zeta_{\text{uncorrelated}}$), that encompasses noise from various other sources including motion artifact, instrumentation noise, thermal noise, and biological sources of noise [194–196]. This extrinsic noise is added to the simulated neural responses (membrane potentials for different neuron types, denoted collectively by **x**):

(74)
$$x^{\prime }(t)=x(t)+\zeta_{\text{uncorrelated}}(t)+\zeta_{\text{correlated}}(t)$$

Since these noise sources are uncorrelated with the responses of the neurons, the power spectrum of the measured signals ${𝓢}^{\prime }(\omega )$ is the sum of the spectrum of the neurons’ activity 𝓢(ω) and the spectra of these noise components:

(75)
$${𝓢}^{\prime }(\omega )=𝓢(\omega )+{𝓢}_{\text{uncorrelated}}(\omega )+{𝓢}_{\text{correlated}}(\omega )$$

The correlated noise is modeled to produce a $\frac{1}{\omega^{4}}$ power law scaling, which is achieved by doubly low-pass filtered white noise. Its power spectrum for each element is given by:

(76)
$${𝓢}_{\text{correlated, element}}(\omega )=\frac{\Sigma_{\text{corr}}}{{\left(1+\tau_{\text{noise}}^{2}\omega^{2}\right)}^{2}}$$

The uncorrelated power spectrum ${𝓢}_{\text{uncorrelated}}(\omega )$ is diagonal, with each diagonal term having a similar filtered form:

(77)
$${𝓢}_{\text{uncorrelated}}(\omega )=D\left(\frac{\Sigma_{\text{uncorr}}}{{\left(1+\tau_{\text{noise}}^{2}\omega^{2}\right)}^{2}}\right)$$

In our simulation, the ratio of power spectral densities of the correlated noise to the uncorrelated noise (i.e, $\Sigma_{\text{corr}}/\Sigma_{\text{uncorr}}$ for corresponding element) is set to 10 for all the results. The noise parameters used in the simulation are as follows,

**Table 3:** Noise parameters

$\tau_{x}$	$\tau_{f}$	$\tau_{\text{noise}}$	$\sigma_{f}$	$\Sigma_{\text{corr}}$	$\Sigma_{\text{uncorr}}$
1 ms	1 ms	50 ms	0.01	9 × 10^−4^	9 × 10^−5^
