# Supplementary material for: Hierarchical Neural Circuit Theory of Normalization and Inter-areal Communication
Source: bioRxiv. 2025 Jul 19:2025.07.15.664935. Preprint. [Version 1] doi: 10.1101/2025.07.15.664935 (PMC12338607; doi:10.1101/2025.07.15.664935)
Supplement: 1 [file NIHPP2025.07.15.664935v1-supplement-1.pdf]

bioRxiv preprint doi: https://doi.org/10.1101/2025.07.15.664935; this version posted July 19, 2025. The copyright holder for this preprint (which was not certified by peer review) is the author/funder, who has granted bioRxiv a license to display the preprint in perpetuity. It is made available under aCC-BY 4.0 International license.

Supplementary Material

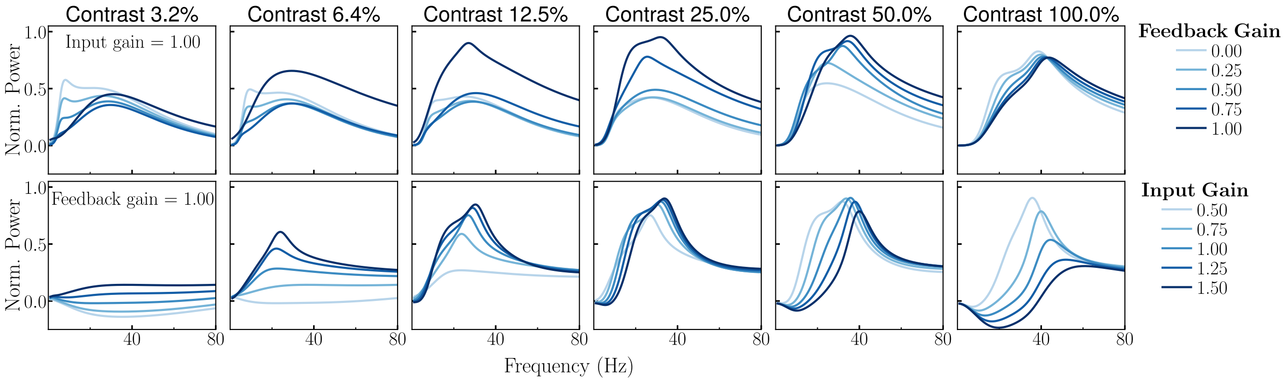

Figure 8: ***Theoretical predictions of varying feedback and input gain on power spectra across different contrast levels.*** *Each column displays results for a specific contrast, with contrast increasing from left to right across panels.* ***Top row,*** *Normalized power as a function of frequency, for varying feedback levels (indicated by the blue line color intensity), with input gain kept at baseline (1.0).* ***Bottom row,*** *Normalized power as a function of frequency for varying input gain levels, with feedback gain kept at baseline (1.0).*

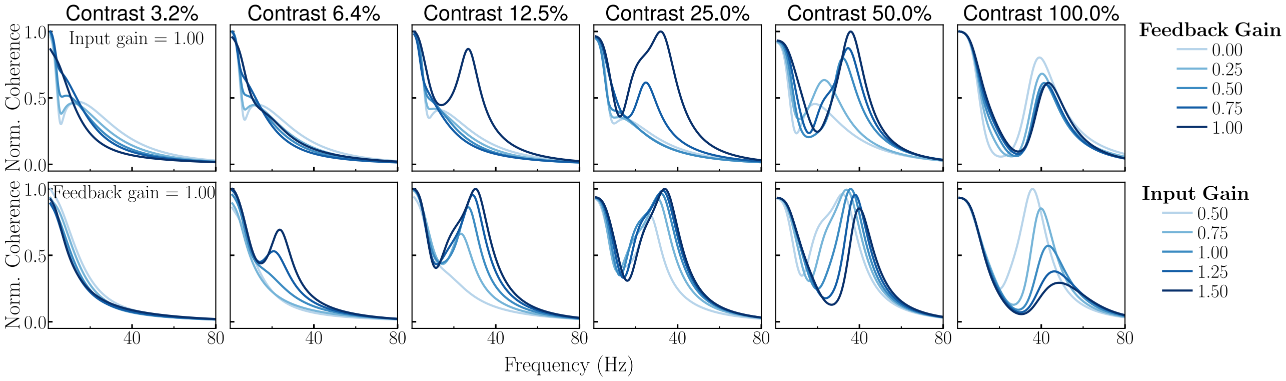

Figure 9: ***Theoretical predictions of varying feedback and input gain on V1-V2 coherence across different contrast levels.*** *Each column displays results for a specific contrast, with contrast increasing from left to right across panels.* ***Top row,*** *Normalized coherence as a function of frequency, for varying feedback levels (indicated by the blue line color intensity), with input gain kept at baseline (1.0).* ***Bottom row,*** *Normalized coherence as a function of frequency for varying input gain levels, with feedback gain kept at baseline (1.0).*

bioRxiv preprint doi: https://doi.org/10.1101/2025.07.15.664935; this version posted July 19, 2025. The copyright holder for this preprint (which was not certified by peer review) is the author/funder, who has granted bioRxiv a license to display the preprint in perpetuity. It is made available under aCC-BY 4.0 International license.

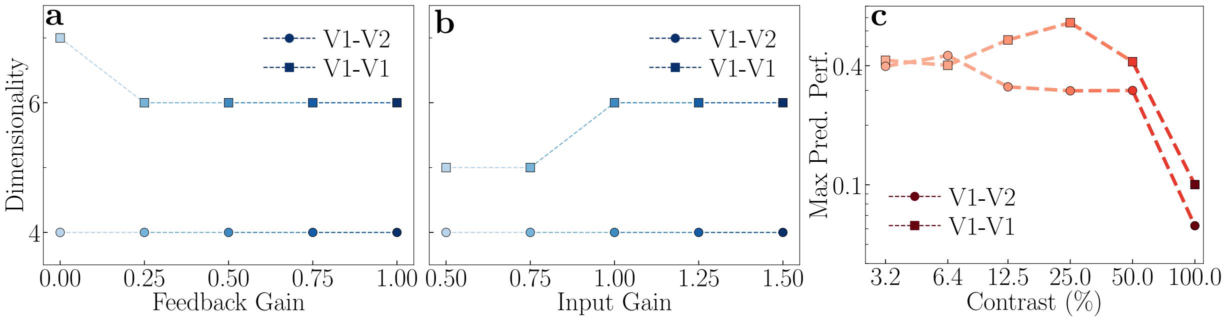

Figure 10: ***Theoretical predictions: Varying feedback gain, input gain, and contrast on communication subspaces. a,*** *Dimensionality of inter-areal (V1-V2, circles) and within-area (V1-V1, squares) communication subspaces as a function of feedback gain, at 100% stimulus contrast.* ***b,*** *Dimensionality of inter-areal (V1-V2, circles) and within-area (V1-V1, squares) communication subspaces as a function of input gain, at 100% stimulus contrast.* ***c,*** *Maximum prediction performance for inter-areal (V1-V2, circles) and within-area (V1-V1, squares) communication as a function of stimulus contrast.*

bioRxiv preprint doi: https://doi.org/10.1101/2025.07.15.664935; this version posted July 19, 2025. The copyright holder for this preprint (which was not certified by peer review) is the author/funder, who has granted bioRxiv a license to display the preprint in perpetuity. It is made available under aCC-BY 4.0 International license.

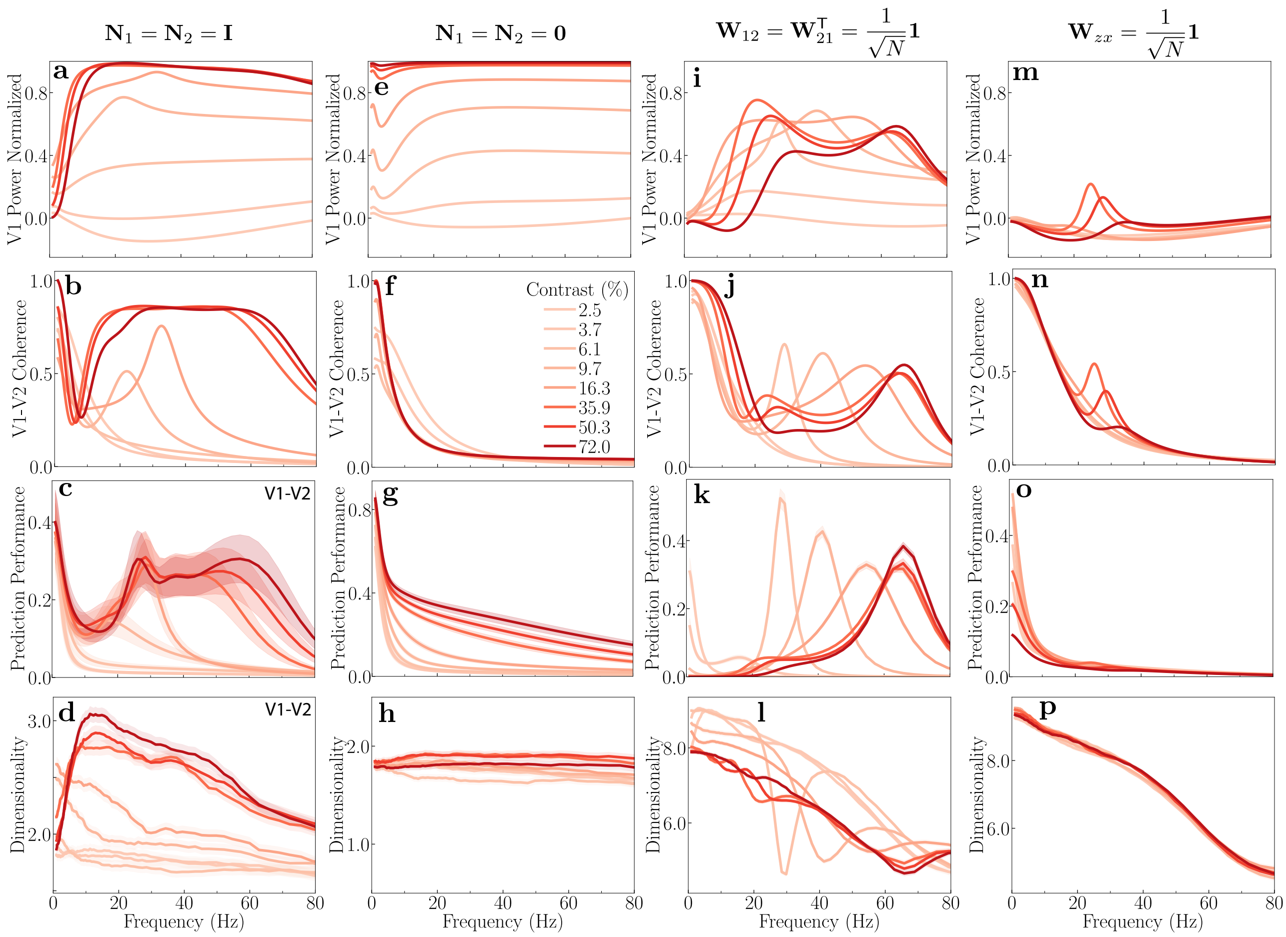

Figure 11: ***Effect of normalization and V1-V2 connectivity on oscillatory activity and communication subspaces.*** *The impact of divisive normalization and inter-area connectivity on network dynamics is explored across four model configurations (columns). For each model, we plot the V1 power spectrum, V1-V2 coherence, prediction performance, and the dimensionality of the communication subspace (rows). For communication subspace analysis, 18 out of 72 simulated neurons were randomly selected from each area, and the analysis was repeated multiple times; shaded areas represent the standard error of the mean (SEM). The number of neurons for the communication subspace analysis was selected for direct comparison with published experimental measurements of communication subspaces with broadband (natural image) input stimuli (see Suppl. Fig. 7 of Ref. [32]).* ***a-d, Self-normalization****: Model predictions when the normalization matrix is set to identity ($\mathbf{I}$), meaning each neuron only normalizes its own activity.* ***a,*** *Normalized V1 power spectra at different stimulus contrast values.* ***b,*** *The coherence peaks between V1 and V2 are narrow band at low contrast, and become broadband at high contrasts ($> 16\%$).* ***c,*** *Prediction performance is highest at frequencies corresponding to peak coherence.* ***d,*** *The communication subspace dimensionality is low, but increases with contrast.* ***e-h, No-normalization****: Model predictions when the normalization matrix is a null matrix (i.e, normalization is removed).* ***e,f*** *Removing normalization eliminates prominent oscillatory activity in both the power and coherence spectra.* ***g,*** *Removing normalization also eliminates peaks in prediction performance.* ***f,*** *Subspace dimensionality remains low and shows little or no dependence on frequency or contrast.* ***i-l, Uniform inter-area connectivity****: Model prediction when the V2 to V1 connectivity matrix modified to $\mathbf{I} + \frac{1}{\sqrt{N}}\mathbf{1}$, where N is number of principal neruons and $\mathbf{1}$ is an ($N \times N$) matrix of ones.* ***i,*** *V1 power shows distinct peaks in the low and high gamma frequency range.* ***j-k,*** *High coherence corresponds to high prediction performance.* ***l,*** *The dimensionality is higher compared to when inter-area connectivity is sparse, but still much lower than the number of neurons (18), and still markedly reduced at frequencies where coherence is strongest.* ***m-p, Uniform external input****: V1 tuning curves are modified to be flat, signifying an equal preference for all stimulus orientations (or equivalently, that the input stimulus is broadband, e.g., noise or natural images).* ***m,n,*** *Oscillatory activity is evident in both power and coherence spectrum.* ***o,*** *Prediction performance decreases with frequency.* ***p,*** *Communication subspace dimensionality is higher compared to when the tuning curves (or stimulus) is narrow band, but still much lower than the number of neurons (18).*
